# Effects of TORC1 Inhibition during the Early and Established Phases of Polycystic Kidney Disease

**DOI:** 10.1371/journal.pone.0164193

**Published:** 2016-10-10

**Authors:** Michelle H. T. Ta, Kristina G. Schwensen, Sheryl Foster, Mayuresh Korgaonkar, Justyna E. Ozimek-Kulik, Jacqueline K. Phillips, Anthony Peduto, Gopala K. Rangan

**Affiliations:** 1 Michael Stern Translational Laboratory for Polycystic Kidney Disease, Centre for Transplant and Renal Research, Westmead Institute for Medical Research, University of Sydney, Sydney, Australia; 2 Department of Radiology, University of Sydney at Westmead Hospital, Sydney, Australia; 3 Faculty of Health Sciences, University of Sydney, Sydney, Australia; 4 Brain Dynamics Centre, Westmead Institute for Medical Research, University of Sydney, Sydney, Australia; 5 Department of Biomedical Science, Faculty of Medicine and Health Sciences, Macquarie University, Sydney, Australia; 6 Department of Renal Medicine, Westmead Hospital, Western Sydney Local Heath District, Westmead, Sydney, Australia; UCL Institute of Child Health, UNITED KINGDOM

## Abstract

The disease-modifying effects of target of rapamycin complex 1 (TORC1) inhibitors during different stages of polycystic kidney disease (PKD) are not well defined. In this study, male Lewis Polycystic Kidney Disease (LPK) rats (a genetic ortholog of human *NPHP9*, phenotypically characterised by diffuse distal nephron cystic growth) and Lewis controls received either vehicle (V) or sirolimus (S, 0.2 mg/kg by intraperitoneal injection 5 days per week) during the early (postnatal weeks 3 to 10) or late stages of disease (weeks 10 to 20). In early-stage disease, sirolimus reduced kidney enlargement (by 63%), slowed the rate of increase in total kidney volume (TKV) in serial MRI by 78.2% (LPK+V: 132.3±59.7 vs. LPK+S: 28.8±12.0% per week) but only partly reduced the percentage renal cyst area (by 19%) and did not affect the decline in endogenous creatinine clearance (CrCl) in LPK rats. In late-stage disease, sirolimus reduced kidney enlargement (by 22%) and the rate of increase in TKV by 71.8% (LPK+V: 13.1±6.6 vs. LPK+S: 3.7±3.7% per week) but the percentage renal cyst area was unaltered, and the CrCl only marginally better. Sirolimus reduced renal TORC1 activation but not TORC2, NF-**κ**B DNA binding activity, *CCL2* or *TNF****α*** expression, and abnormalities in cilia ultrastructure, hypertension and cardiac disease were also not improved. Thus, the relative treatment efficacy of TORC1 inhibition on kidney enlargement was consistent at all disease stages, but the absolute effect was determined by the timing of drug initiation. Furthermore, cystic microarchitecture, renal function and cardiac disease remain abnormal with TORC1 inhibition, indicating that additional approaches to normalise cellular dedifferentiation, inflammation and hypertension are required to completely arrest the progression of PKDs.

## Introduction

The mammalian target of rapamycin complex 1 (TORC1) is an important promoter of cell growth and cyclin D1/pRb activation, and is over-activated in response to mutational dysfunction of cilia-associated proteins in polycystic kidney disease (PKD) [1], [2], [3] [4]. In preclinical studies, small molecule inhibitors of TORC1 have consistently reduced kidney enlargement and cyst growth in genetically and non-genetically orthologous animal models of PKD [5], [6], [7], [8]. However, in clinical trials of autosomal dominant PKD (ADPKD), the therapeutic efficacy of TORC1 inhibitors (everolimus, sirolimus) has not been confirmed [9, 10]. For example, Walz et al. found that in patients with established ADPKD and renal impairment [mean total kidney volume (TKV) of 1911 ml; estimated glomerular filtration (eGFR) 30–89 ml/min/1.73 m^2^], treatment with everolimus for 2 years slowed the progression of kidney enlargement but worsened the estimated GFR (eGFR) [10]. On other hand, Serra et al. reported that in ADPKD patients with established kidney enlargement (median TKV of 1003 ml) and preserved renal function, treatment with sirolimus for 18 months did not halt kidney growth [9].

Two hypotheses have been proposed for the inconsistency between human and animal studies: (i) there are inter-species variations in the bioavailability and/or dose of TORC1 inhibitors required to suppress kidney cyst growth *in vivo* [11]; (ii) TORC1 inhibitor efficacy is critically dependent on the duration as well as the timing of commencing treatment in relation to kidney enlargement [8]. Regarding the latter, the majority of preclinical studies using TORC1 inhibitors may have achieved suppressive effects on renal cyst growth because treatment was initiated prior to the peak in TKV or the time of maximal cystic epithelial cell (CEC) proliferation [5] [6, 12], [8], [7]. Indeed, in some animal models, the expression of TORC1 and cell cycle proteins as well as CEC proliferation exhibit time-dependent changes [13, 14], suggesting that there might be a therapeutic window in which anti-proliferative inhibitors are most effective in preventing kidney enlargement in certain types of PKDs [13]. Another proposed mechanism by which sirolimus could reduce kidney enlargement is the regression of renal cyst growth [7, 8], but the underlying mechanisms and therapeutic significance of this are not certain. In addition the effects of TORC1 inhibitors on other aspects of chronic renal injury associated with PKD have received little attention. In non-PKD animal models of chronic kidney disease, TORC1 inhibition has anti-inflammatory and anti-fibrotic effects in the interstitium [15, 16] and this is also relevant to PKD [17]. Moreover, the effects on renal function, cilia morphology and cardiovascular disease have not been fully assessed in previous preclinical studies [18].

To better understand the efficacy of TORC1 inhibition in PKD, in the present study we compared the effects of sirolimus on renal cyst enlargement, interstitial injury, renal function and cardiovascular disease when initiated during the early and established stages of disease in Lewis Polycystic Kidney (LPK) rats. The LPK rat is genetically orthologous to human *NPHP9* in which the early phase of disease (postnatal weeks 3 to 10) is characterised by synchronised diffuse distal nephron cystic growth whereas the established stage also includes additional features of further decline in renal impairment, accompanying renal tubulointerstitial disease and hypertension, and eventually the development of terminal end-stage kidney disease after week 20 [19]. Thus, the LPK rat model provides an opportunity to thoroughly evaluate the effects of sirolimus during various disease stages. In this study, we hypothesised that the timing of sirolimus initiation is an important determinant in attenuating kidney enlargement in the LPK rat model and that early commencement of drug (weeks 3 to 10) might be more effective in reducing kidney enlargement but that late initiation of treatment (weeks 10 to 20) would still be associated with improvements in interstitial fibrosis, renal function and hypertension and promote cyst regression.

## Materials and Methods

### Animals

Animals were housed under standard conditions (artificial lighting; light:dark cycle 1800–0600 hrs) at the animal facility in the Institute of Clinical Pathology and Medical Research (ICPMR; Westmead Hospital) and allowed food and water *ad libitum*. LPK rats and Lewis/SSN rats were obtained from the breeding colony at Westmead Hospital [20]. All protocols and procedures were approved by the Animal Ethics Committee, Westmead Hospital, Western Sydney Local Heath District (Protocol Number 4100), and conducted according to the Australian Code for the Care and Use of Animals for Scientific Purposes [21].

### Experimental Design

Three studies were undertaken: (i) Study 1: To determine the time-dependent changes in the expression of downstream targets for TORC1 (phosphorylated S6 ribosomal protein and 4E-BP1) and TORC2 (phosphorylated Akt) in male Lewis and LPK rats with disease progression, kidney tissue was examined at postnatal weeks 1, 3, 6, 10, 16 and 20 (n = 3 for Lewis and n = 6 for LPK per timepoint) using archival tissue from another experiment [22]; (ii) Study 2: To determine the effects of early treatment with sirolimus on renal cystic disease in LPK rats, three week old male rats received either the vehicle or sirolimus (0.2 mg/kg per day by intraperitoneal injection for 5 days per week) from week 3 until week 10 (n = 9 for LPK+sirolimus, n = 11 for LPK+vehicle). Age-matched male Lewis animals received either sirolimus or vehicle (n = 4 per group). For electron microscopy, kidney tissue from an additional two male LPK rats aged 10 weeks was used as control tissue for this cohort; (iii) Study 3: To determine the effects of late treatment with sirolimus on renal cystic disease in LPK rats, ten week old males were treated with either vehicle or sirolimus (0.2 mg/kg per day by intraperitoneal injection for 5 days per week) from week 10 until week 20 (n = 10 per group). Age-matched Lewis animals received either sirolimus or vehicle (n = 5 per group).

At the endpoint of each study, all rats received a single injection of bromodeoxyuridine (BrdU, 50 mg/kg dissolved in sterile normal saline) three hours prior to euthanasia to label proliferating cells in the kidney. At the time of tissue collection, rats were anaesthetised by an intraperitoneal injection of ketamine xylazine, a mid-line laparotomy was performed, blood was collected, and heart and kidneys were removed and weighed, and then used for histological (immediately placed in fixation solution) and mRNA analysis (immediately snap-frozen in liquid nitrogen and then stored at -70 C).

Sirolimus was purchased from LC Laboratories (MA, USA) and prepared as a stock solution (50 mg/ml in DMSO) and stored at -20 C. The stock solution was diluted (0.25 mg/ml) in vehicle (20% DMSO, 20% ethanol, 60% normal saline for injection, v/v), filter-sterilised and stored at 4 C until the time of injection. The dilutions were prepared and drawn into insulin syringes daily according to the body weight of each animal. The optimal dose and route of sirolimus administration was determined in two pilot studies. Briefly, in the first pilot, treatment with sirolimus in drinking water (Rapamune, Pfizer; 1mg/ml; 2 mg/kg/day) for 8 weeks in LPK rats only partly attenuated kidney enlargement and whole blood levels of sirolimus were variable. In the second pilot, a dose-finding study showed subcutaneous injections of sirolimus (obtained from LC Laboratories) at 0.2 mg/kg/day was well tolerated and reduced kidney enlargement over 1 week (data not shown). Because, s.c. injections caused superficial ulceration in some rats, the intraperitoneal route was chosen.

### Assessment of renal function

Serum collected from blood in Studies 2 and 3 was analysed for urea, creatinine, albumin and cholesterol as described previously [23]. Sirolimus levels were measured in whole blood by an immunoassay (Mr John E. Ray, Department of Clinical Pharmacology, St Vincent’s Hospital, Sydney, Australia). To determine proteinuria and endogenous creatinine clearance (CrCl), individual rats were placed in metabolic cages for 16 hours to collect urine. To minimise the time spent in the metabolic cages and chronic stress, rats were not acclimatised to the metabolic cages prior each urine collection, as required in the Animal Ethics Protocol (No. 4100). Urinary protein and creatinine, and calculation of the CrCl, were determined according to previous methods [23].

### Assessment of total kidney volume by magnetic resonance imaging (MRI) using a clinical 3T scanner

The progression of total kidney enlargement was assessed by two methods: (i) measurement of the two kidney weight corrected for the body weight at the end of the experiment; and (ii) measurement of the total kidney volume by MRI performed on a subset of randomly selected animals in Studies 2 and 3, using techniques described previously [20] and in the S1 File (S1 Fig and S2 Fig). In Study 2, a total of 33 MRI scans were performed in 20 animals (Lewis+vehicle, n = 3; Lewis+sirolimus n = 3; LPK+vehicle, n = 8, LPK+sirolimus, n = 6) at postnatal weeks 4/5 (1 week after commencing treatment; denoted the Baseline scan), week 6 (3 weeks after commencing treatment) and weeks 9/10 (7 weeks after commencing treatment; denoted the End-of-Treatment scan). In Study 3, a total 16 scans were performed in 8 animals (LPK+vehicle, n = 4; LPK+sirolimus, n = 4) at postnatal week 10 (within 1 week of commencing treatment; denoted the Baseline scan) and at week 17 (7 weeks of commencing treatment, denoted the End-of-Treatment scan).

### Renal and cardiac histology, immunohistochemistry and quantitation

Mid-coronal slices of kidney or heart were immersion-fixed in either 10% formalin or methyl Carnoy solution for 12 hours prior to tissue processing. Sections 4–6 microns in thickness were deparaffinised and then either stained by periodic acid-Schiff (PAS), Sirius-red or used in immunohistochemistry. The latter was performed as previously described [24] using primary antibodies against ED-1 (1:400, MCA341R; Serotec, Kidlington, U.K.), BrdU (1:100, clone MoBu-1, Novus Biologicals, Littleton, CO, USA), Ki-67 (1:100, clone SP6, Lab Vision, CA, USA) and α-SMA (1:4000, A2547; Sigma–Aldrich, St. Louis, MO, USA) to assess for CD68-positive monocytes, cell proliferation (both BrdU and Ki-67) and myofibroblasts/vascular smooth muscle cells respectively.

To determine the effects of sirolimus on TORC1 and TORC2, activation, the expression of downstream targets was assessed by immunohistochemistry using the following primary antibodies: (i) polyclonal anti-rat rabbit phosphorylated S6 ribosomal protein (Ser235/236) (p-S6) (1:150; #2211, Cell Signalling Technology, Danvers, MA, USA); (ii) anti-rat rabbit phosphorylated eukaryotic translation initiation factor 4E-binding protein 1 (p-4E-BP1) (1:1000; clone 236B4, Cell Signalling Technology, Danvers, MA, USA); (iii) polyclonal rabbit anti-rat phosphorylated Akt (Ser/Thr) (p-Akt) (1:200; #9611, Cell Signalling Technology, Danvers, MA, USA). Immunohistochemistry for p-S6, p-4E-BP1 and p-Akt was performed by antigen retrieval (by 10 minutes of microwave oven heating), overnight incubation with the primary antibody, application of secondary biotinylated antibodies, followed by immunodetection with diaminobenzidine on methylgreen counterstained slides [24].

For quantitative histological analysis of renal disease, slides were digitised with a whole-slide scanner (Scanscope CS2, Leica Microsystems, North Ryde, Australia) and image analysis for percentage cystic area and positive immunostaining was performed using Imagescope software (Version 13) as described in previous studies [13], [24].

### Assessment of renal NF-κB activation and κB-dependent inflammatory genes

The renal expression of NF-**κ**B activation and **κ**B-dependent inflammatory genes (*TNF***α** and *CCL2*) was used to determine the effects of sirolimus on inflammatory signalling. NF-**κ**B activation was assessed in renal cortical nuclear extracts using a p65 transcription factor assay kit (100007889; Cayman Chemical, Ann Arbor, MI, USA) and by immunodetection of p105/50 (1:100, P19838, Epitomics, Burlingham, CA, USA) in formalin-fixed slides, as previously described [24]. The technique for quantifying renal *TNF***α** and *CCL2* is provided in the S1 File [25–27].

### Assessment of cilia ultrastructure by electron microscopy

Following euthanasia, kidneys were collected, sectioned into coronal slices 1mm thick, and fixed in 2.4% glutaraldehyde, 2% paraformaldehyde in MOPS buffer [3-(N-morpholino) propanesulfonic acid, sodium acetate, EDTA; pH 7.2]. For scanning electron microscopy (SEM), samples were sectioned into 1mm thick wedges, washed in 0.1M PB, post-fixed in 1% osmium tetroxide solution, washed in 0.1M PB, dehydrated in graded series of ethanol (30–100%) and critical point dried in EMITECH K850 Critical Point drier, with argon as a transition fluid. Kidney fragments were mounted on the aluminium stubs, covered previously with carbon tabs, with a surface of the section facing upwards, and coated with gold using EMITECH sputter coater K550. Samples were viewed with JEOL JSM-6480 LA scanning electron microscope at Macquarie University (Jeol USA, Inc., MA, USA) and files stored as jpeg image format. Images were then used to determine cilia length. Cilia were selected from random fields of distal and collecting tubules. The collecting tubule was identified by the presence of the intercalated cells, and distal tubules by a lack of the dark cells and brush border, short microvilli, and distinctive separation of the cells from the adjacent ones.

### Assessment of cardiovascular disease

Tail arterial systolic blood pressure was measured noninvasively in conscious rats by piezoplethysomography using a tail sensor and tail-cuff inflation (MacLab, ADInstruments, Bella Vista, Australia). Systolic blood pressure was defined as the appearance of the tail arterial pulse wave with cuff deflation. Rats were acclimatised to the method of restraint during the blood pressure determination, and the mean of five measurements at one session was obtained for each animal at a particular timepoint. Cardiac disease was also assessed by the heart weight corrected for body weight at the time of tissue collection, and in PAS- and Sirius red-stained sections. The cardiac expression of phosphorylated -S6 and -Akt was assessed in formalin-fixed sections, as described earlier.

### Statistics and Data Analysis

Data are presented as mean±SD, and were analysed with JMP (version 4.04, SAS institute, Carey, NC, USA) and GraphPad Prism (La Jolla, CA, USA). Comparisons between the experimental groups were performed by one-way analysis of variance (ANOVA), followed by a post-hoc analysis with the Tukey–Kramer HSD test. A P-value of <0.05 indicated statistical significance. For PCR data, the Kruskal-Wallis one-way ANOVA was applied, with appropriate post-hoc tests. Two animals in Study 2 died (both from the LPK+vehicle) at the time of anaesthesia during MRI scans due to respiratory depression and cardiac arrest, and only data to calculate TKV at week 4 (n = 1 each) and week 6 (n = 1) is included in the Results. All other animals in Study 2 (Lewis n = 8, LPK n = 18) survived for the duration of the study.

## Results

### Time-course of renal TORC1 and TORC2 activation in LPK rats

**Fig 1:**
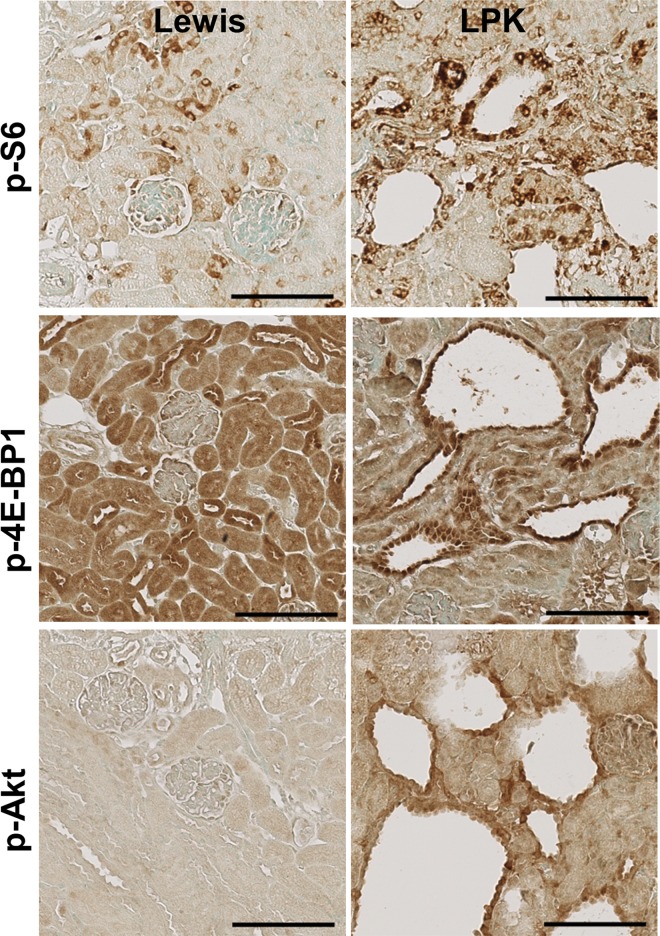
Renal expression of p-S6, p-4EBP1 and p-Akt in Study 1. Representative photomicrographs showing immunostaining for p-S6, p-4EBP1 and p-Akt in the kidney cortex of Lewis and LPK rats at week 3. Scale bar = 100μm.

**Fig 2:**
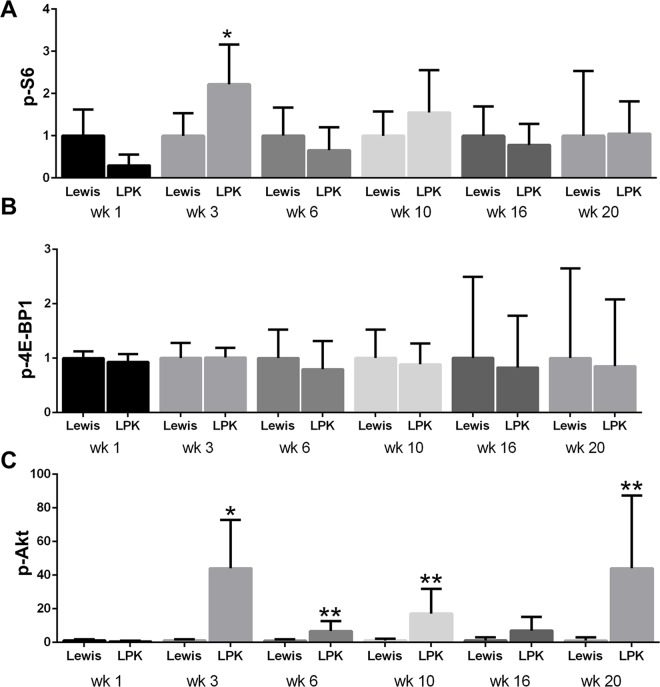
Quantitative analysis of renal p-S6, p-4E-BP-1 and p-Akt immunostaining in Study 1. A. Time-course of p-S6 expression; B. Time-course of p-4EBP1 expression; C. Time-course of p-Akt expression. Data are expressed as mean±SD; *P<0.05 vs. Lewis for the corresponding time-point; **P<0.01 vs. Lewis for the corresponding timepoint; n = 3 rats per time-point for Lewis and n = 6 rats per timepoint for LPK.

The time-dependent changes in the expression and localisation of TORC1 and TORC2 targets (p-S6, p-4E-BP1 and p-Akt) were first assessed in untreated Lewis and LPK rats (Study 1). In Lewis rats, the expression of p-S6 in the kidney was weak and diffuse in the cytoplasm of tubular epithelial cells in the cortex (Fig 1, upper panel) and more intense in tubules in the outer medulla. This pattern of localisation remained consistent from weeks 1 to 20. In kidneys from LPK rats, staining for p-S6 was increased in comparison to the Lewis group, and was particularly strong in epithelial cells lining cysts, distal tubules and in interstitial cells of the cortex and inner medulla (Fig 1, upper panel). By quantitative analysis of whole-slide images, the expression of p-S6 fluctuated over the 20 week time period, but the peak increase in p-S6 in LPK rats occurred at week 3 (Fig 2).

In Lewis rats, p-4E-BP1 was strongly expressed in cortical tubules and moderate in medullary tubules, and this pattern of expression remained consistent from weeks 1 to 20 (Fig 1, middle panel). In LPK kidneys, at weeks 1 and 3, p-4E-BP1 was strongly expressed in dilated distal tubules and cyst-lining epithelial cells, and diffuse in the cortical interstitium. From week 6 onwards, interstitial staining was less prominent compared to previous time-points, while the immunopositivity in cystic epithelial cells remained consistent. However, by quantitative analysis of whole-slide images, the overall expression of p-4E-BP1 in LPK rats was similar to the Lewis group at all time points (Fig 2).

In Lewis rats, the expression of p-Akt expression was weak and diffuse, and observed in cortical and medullary tubules (Fig 1, lower panel). In kidneys from LPK rats, p-Akt expression was strongly induced compared to Lewis rats, and detected in renal tubules as well as in cyst-lining epithelial cells (Fig 1, lower panel). From week 10 onwards, the degree of cystic epithelial cell staining was weaker compared to earlier time-points, but greater positivity was observed in the cortical interstitium. By quantitation of whole-slide images, p-Akt was strongly induced in LPK rats compared to Lewis animals at weeks 3, 6, 10 and 20 (Fig 2). Taken together, these data show that the activation of both TORC1 and TORC2 occurs in renal cysts at all time points in LPK rats, as determined by immunodetection of their downstream targets.

### Renal effects of sirolimus when initiated in early-stage PKD

#### Kidney enlargement

**Fig 3:**
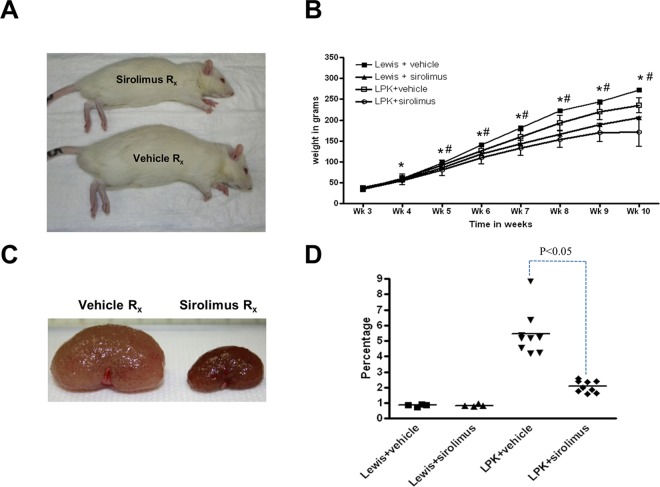
Effects of early initiation of sirolimus on body weight and kidney enlargement (Study 2). Early commencement of sirolimus reduces kidney enlargement in LPK rats. A. Photomicrographs showing effects of sirolimus on body size in LPK rats after seven weeks of treatment; B. Time-course of body weight in the experimental groups from weeks 3 to 10; C. Photomicrographs showing effects of sirolimus on kidney size in LPK rats after seven weeks of treatment; D. Effect of sirolimus on kidney enlargement, as assessed by the percentage two-kidney weight to body weight ratio at week 10. In Panel B, data expressed as mean±SE; *P<0.05 for Lewis+vehicle vs. Lewis+sirolimus at each time-point; ##P<0.05 for LPK+vehicle vs. LPK+sirolimus at each time-point; n = 4 per group for Lewis rats and n = 9 per group for LPK rats.

**Fig 4:**
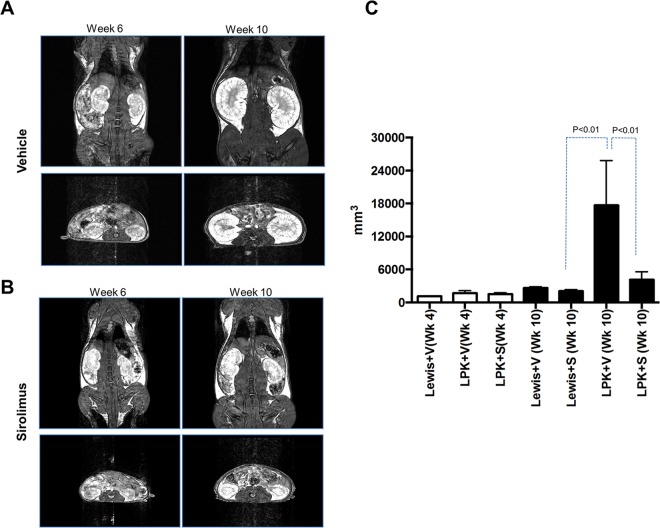
Early initiation of sirolimus reduces the increase in total kidney volume in LPK rats. A and B. Representative MR images of the experimental groups at weeks 6 and 10. An in-plane resolution of approximately 0.28–0.39mm and an effective through-plane resolution of 0.8mm for both the coronal and axial image sets within acceptable scan times (coronal: 6min 44sec and axial: 5min 26sec) was achieved. Signal-to noise ratio was sufficient to enable accurate segmentation and volume calculation using 3D SLICER. Anatomical detail of the kidneys was well visualized. Images from the 3D FIESTA sequence are shown. C. Graph showing total kidney volume (TKV) in the experimental groups at weeks 4 and 10. The TKV increased by ~2.4 times in both the Lewis+vehicle and LPK+sirolimus groups from weeks 4 to 10. In contrast in the LPK+vehicle group increased by 11.6 times over the same period. Data expressed as mean±SD; At week 4, Lewis+vehicle: n = 1; LPK+vehicle:; LPK+sirolimus:; n = 3–4 per group) and at week 10, Lewis+vehicle n = 3; Lewis+sirolimus n = 3; LPK+vehicle n = 6; LPK+sirolimus.

Treatment with sirolimus from week 3 to 10 attenuated the growth of LPK and Lewis rats compared to vehicle-treated animals (p<0.05) (Fig 3). Kidney enlargement, as determined by the kidney to body weight ratio at the end of the study (week 10), was reduced by 63% in LPK rats compared to the vehicle (P<0.05, Fig 3). By MRI, the baseline scan (week 4) showed that TKV was similar in both LPK groups (Lewis+vehicle: 1105 mm^3^ n = 1; LPK+vehicle: 1518±246 mm^3^ and LPK+sirolimus: 1669±455 mm^3^; both n = 3–4 per group). By the mid-point scan at week 6, the treatment groups were starting to differ (Lewis+vehicle: 1508 mm^3^, n = 1; LPK+vehicle: 3024±391 mm^3^, n = 2; LPK+sirolimus: 1890±228 mm^3^, n = 2) and by the end of treatment at week 10, sirolimus significantly reduced the increase in TKV in LPK rats compared to vehicle (Lewis+vehicle: 2644±215 mm^3^, n = 3; Lewis+sirolimus: 2066±264 mm^3^, n = 3; LPK+vehicle: 17670±8141, n = 6; LPK+sirolimus: 4143±1437 mm^3^, n = 6; P<0.05; Fig 4). To evaluate the relative treatment effect of sirolimus, we calculated the rate of change in TKV from the baseline to end-of-treatment MRI scans (expressed as a percentage increase in TKV per week) in LPK rats, and found that this was reduced by 78.2% with sirolimus treatment (LPK+vehicle: 132.3±59.7 vs. LPK+sirolimus: 28.8±12.0% per week; P<0.05).

#### Renal Function

The serum creatinine increased in the LPK+vehicle group compared with Lewis+vehicle but was unchanged by sirolimus treatment (Table 1). Creatinine clearance was 59.0% lower in LPK+vehicle compared to Lewis+vehicle (Table 1), and this was also not altered by sirolimus treatment. Unexpectedly, sirolimus reduced CrCl by 42.4% in Lewis animals (p = 0.02 vs. compared to Lewis+vehicle). There were no changes in serum urea, creatinine or albumin with sirolimus treatment in either Lewis or LPK rats. Whole blood sirolimus, measured 4 hours after the last injection, was detected in all treated animals (Table 1).

**Table 1:** Renal function, serum cholesterol and whole blood sirolimus levels in Lewis and LPK rats at week 10 in the early treatment study (Study 2). Data are expressed as mean±SD; Abbreviations: CrCl, creatinine clearance; *P<0.05 when compared to Lewis+vehicle; ND, not determined.

	Lewis+vehicle	Lewis+sirolimus	LPK+vehicle	LPK+sirolimus
	N = 4	N = 4	N = 9	N = 9
Creatinine (μmol/L)	29±5	32±3	49±9*	53±24*
Urea (mmol/L)	5.4±1.1	5.0±0.7	15.6±4.6	13.1±9.3
CrCl (μL/min/cm^2^)	6.6±0.8	3.8±1.6*	2.7±0.9*	2.9±1.2*
Albumin (g/dL)	30±2	29±1	27±3	31±5
Cholesterol (mmol/L)	1.5±0.2	2.3±0.3*	2.6±0.4*	3.2±0.5*
Sirolimus (ng/ml)	ND	11.8±6.2	ND	15.7±7.5

#### Cystic Renal Disease

At Week 10, renal disease in LPK rats was characterised by diffuse cystic dilatation of the distal nephron accompanied by interstitial inflammation and fibrosis (Table 2 and data not shown). By quantitative analysis, the cross-sectional area of the kidney in the vehicle-treated LPK group was approximately 1.5-fold larger than that of Lewis rats (Table 2). In LPK rats, sirolimus reduced the cross-sectional area of the kidney by 39.8% and the percentage cyst area by 34.3% (Table 2). Treatment with sirolimus reduced the proliferation of cells in the kidney, as determined by the percentage of BrdU+ (by 56.3%) and Ki67+ cells (by 57.4%), compared to the LPK+vehicle group. In LPK rats, sirolimus partially reduced the accumulation of interstitial monocytes and interstitial collagen deposition but did not affect the number of interstitial myofibroblasts (**α**SMA+ cells) (Table 2).

**Table 2:** Effect of sirolimus on cystic renal disease at Week 10 in early-stage PKD. Data are expressed as mean±SD; Abbrevations: BrdU, bromodeoxyuridine; α-SMA, alpha-smooth muscle actin; SR, Sirius-Red; *P<0.05 when compared to Lewis+vehicle; #P<0.05 when compared to LPK+vehicle; ## P<0.01 when compared to LPK+vehicle.

	Lewis+vehicle	Lewis+sirolimus	LPK+vehicle	LPK+sirolimus
	N = 4	N = 4	N = 9	N = 9
Kidney section area (mm^2^)	43.7 ± 3.6	38.5 ± 3.3	65.4 ± 14.4*	39.4 ± 6.0* #
Cystic area (%)	-	-	55.1 ± 5.1	36.2 ± 6.9#
BrdU+ cells (%)	1.7 ± 0.8	1.3 ± 0.6	17.6 ± 7.7*	7.7 ± 3.8##
Ki67+ cells (%)	0.18 ± 0.10	0.15 ± 0.09	1.76 ± 1.11*	0.75 ± 0.47##
ED-1+ cells (%)	23.5 ±5.8	15.8 ± 1.0 *	28.2 ± 2.2	21.7 ± 2.4#
α-SMA+ cells (%)	10.6 ± 0.5	11.1 ± 0.4	19.1 ± 0.3*	21.7 ± 0.9
Interstitial collagen (SR-positive) (%)	4.7 ± 1.1	5.1 ± 1.9	12.1 ± 3.9*	7.7 ± 2.4*#

#### Renal TORC1 and TORC2 activation

**Fig 5:**
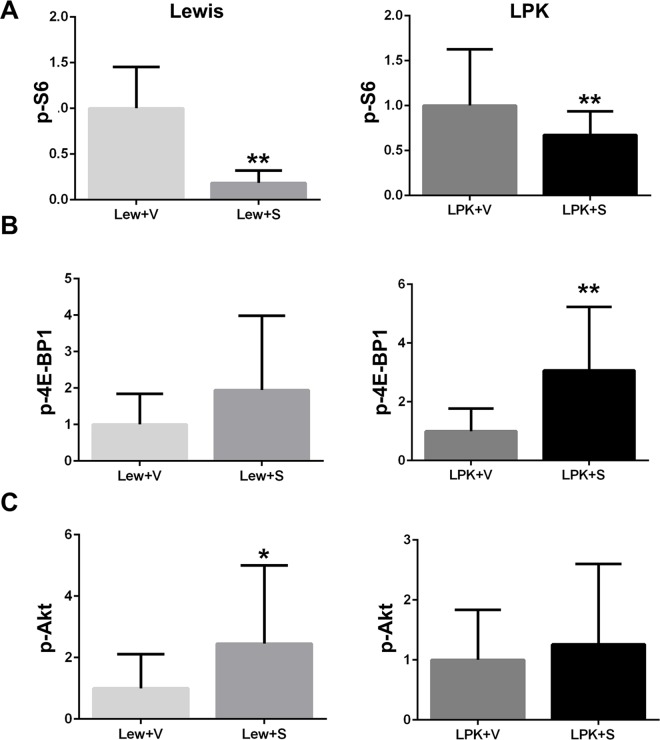
**Effect of early initiation of sirolimus on p-S6 (A), p-4E-BP1 (B) and p-Akt (C), as assessed by quantitative analysis of immunostaining.** Data (mean±SD) are expressed as the fold-change over the average for vehicle-treated animals; *p<0.05 vs. vehicle; **p<0.01 vs. vehicle; n = 4 per group for Lewis rats and n = 9 per group for LPK rats.

**Fig 6:**
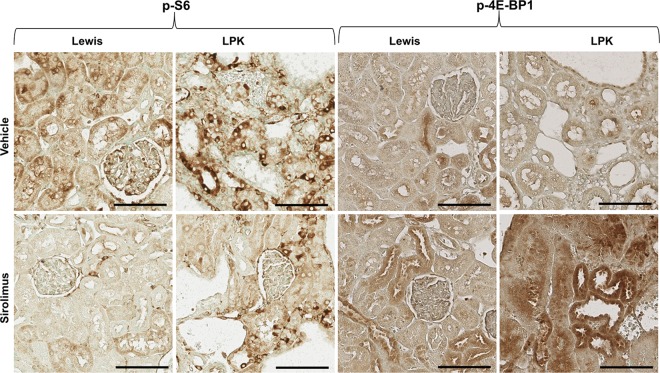
Representative immunohistochemistry images for p-S6 and p-4EBP1 following early initiation of sirolimus. Shown are representative photomicrographs of kidney cortices from Lewis and LPK rats given sirolimus from week 3 until week 10. Scale bar = 100μm.

In Lewis rats, treatment with sirolimus from weeks 3 to 10, reduced the renal expression of p-S6 and increased renal p-Akt, compared to the vehicle group (Figs 5 and 6). In LPK rats, sirolimus partially reduced p-S6 and increased p-4E-BP1 (Figs 5 and 6) but did not affect p-Akt compared to the vehicle group.

#### Renal pro-inflammatory gene expression and NF-κB activation

**Fig 7:**
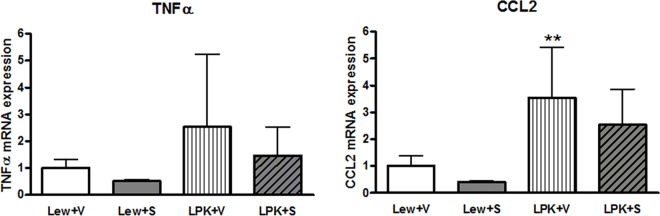
Effects of early initiation of sirolimus on NF-κB dependent proinflammatory gene expression (*TNFα* and *CCL2*) in Study 2. The mRNA expression is shown as the target gene corrected for GAPDH, and expressed as a fold-change over Lewis+vehicle (V). Data are expressed as mean±SD; **p<0.01 vs. Lewis+V; n = 4 per group for Lewis rats and n = 9 per group for LPK rats.

There were no significant differences in *TNFα* expression among the groups (Fig 7), but *CCL2* expression was significantly higher in vehicle-treated LPK rats compared to the Lewis+vehicle group (p<0.01, Fig 7). There was a trend towards a reduction in *CCL2* expression in Lewis+sirolimus vs. Lewis+vehicle (p = 0.10) but this was less discernible in the LPK+vehicle compared to the LPK+sirolimus groups (p = 0.136). In addition, there was no significant effect of sirolimus on nuclear p65 DNA binding activity in either Lewis or LPK rats (Lewis+vehicle: 1.0±0.3, Lewis+sirolimus: 0.8±0.1, LPK+vehicle: 0.8±0.2, LPK+sirolimus: 0.6±0.1; fold-change above Lewis+vehicle, all P>0.05) or in phosphorylated p105 immunostaining (S3 Fig).

### Renal effects of sirolimus when initiated in late-stage PKD

#### Kidney enlargement

**Fig 8:**
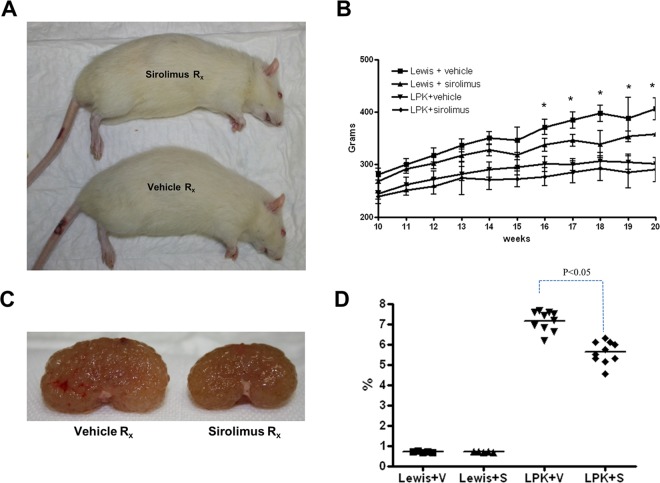
Effect of late initiation of sirolimus on body growth and kidney enlargement in Study 3. A. Representative photomicrographs showing effects of sirolimus on body size; B. Time-course of body weight in the experimental groups from week 10 to week 20; C. Representative photomicrographs of kidney enlargement in LPK rats; D. Effect of sirolimus on the percentage two-kidney weight to body weight ratio at week 20; Data expressed as Mean±SE in panel B, *P<0.05 for Lewis+vehicle vs Lewis+sirolimus; n = 5 per group for Lewis rats and n = 10 per group for LPK rats.

**Fig 9:**
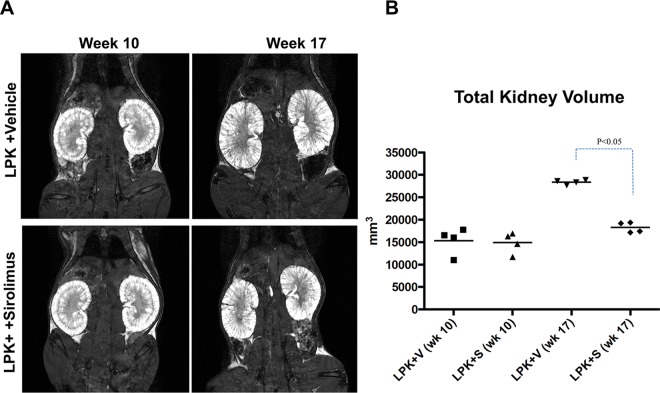
Effect of late initiation of sirolimus on the progression of total kidney volume in LPK rats (Study 3). A. Representative serial MRI scans of LPK rats treated with either vehicle (V) or sirolimus (S) from week 10 to 17. Treatment with sirolimus prevented the increase in total kidney volume (TKV); B. Quantitative analysis of TKV in the experimental groups. The increase in TKV between weeks 10 and 17 was 1.9 times in LPK+V, compared to 1.1 times in the LPK+S group; n = 4 per group per timepoint.

Overall, treatment with sirolimus did not significantly affect body weight in late-stage PKD. Rats treated with sirolimus from postnatal weeks 10 to 20 had a slight reduction in the mean body weight compared to the vehicle groups but this was only statistically significant in Lewis+sirolimus vs. Lewis+vehicle groups from Week 16 onwards (Fig 8). At week 20, kidney enlargement, as determined by the kidney to body weight ratio, was reduced by 22% in LPK rats (P<0.05, Fig 8). By MRI, the TKV at the start of treatment at week 10 was similar in both groups (LPK+vehicle: 15334±2968 mm^3^ vs. LPK+sirolimus: 14892±2330 mm^3^). Towards the end of treatment (week 17), sirolimus reduced TKV by 35% (Fig 9).

Although the rate of increase in TKV in LPK+vehicle rats (13.1±6.6% per week; n = 4), between weeks 10 to 20 was ~10-fold slower in Study 3 compared Study 2 (see above) it was still attenuated to a similar degree (by 71.8%) with sirolimus treatment (LPK+sirolimus: 3.7±3.7% per week; n = 4; P<0.05 compared to LPK+vehicle). On the other hand, the renal microarchitecture, as assessed on the MRI, remained abnormal in sirolimus-treated LPK rats (S4 Fig).

#### Renal Function

**Fig 10:**
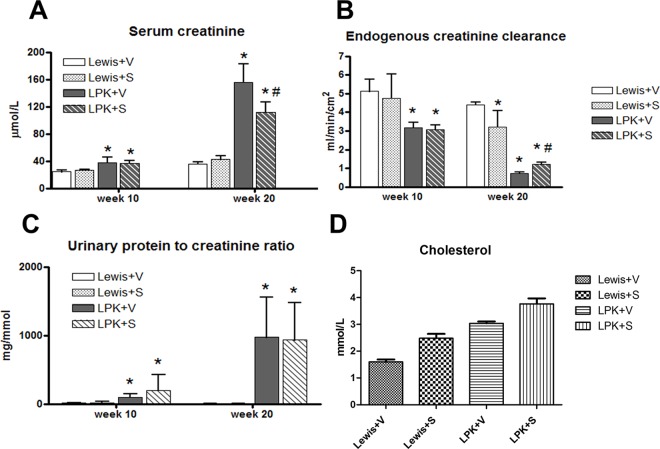
Effect of late initiation of sirolimus on the progression renal dysfunction and proteinuria at baseline (week 10) and at the end of treatment (week 20) (Study 3). A. Serum creatinine; B. Endogenous creatinine clearance; C. Urinary protein to creatinine ratio; D. Serum cholesterol. Data are expressed as mean±SD; *P<0.05 vs. Lewis+V; #P<0.05 vs. LPK+V; n = 5 per group for Lewis rats and n = 10 per group for LPK rats.

The increase in urine volume in LPK rats was not affected by sirolimus (Week 10: Lewis+vehicle: 5±1, Lewis+sirolimus: 4±2, LPK+vehicle: 13±5, LPK+sirolimus: 12±4 ml/16h; Week 20: Lewis+vehicle: 5±1, Lewis+sirolimus: 5±2, LPK+vehicle: 21±4, LPK+sirolimus: 25±5 ml/16h). Renal function, as determined by the serum creatinine, CrCl and serum urea was partially improved by sirolimus in LPK rats (Fig 10; Serum Urea: Week 20: Lewis+vehicle: 6.0±0.5, Lewis+sirolimus: 6.0±0.8, LPK+vehicle: 39.0±3.0, LPK+sirolimus: 31.0±5.0 mmol/L; P<0.05 for LPK+vehicle vs LPK+sirolimus). The urinary protein to creatinine ratio (Pr:Cr) was elevated in LPK animals compared to Lewis rats at baseline (week 10) and at the end of treatment (week 20) (Fig 10). Sirolimus did not alter the urine Pr:Cr ratio in Lewis or LPK animals (Fig 10), but increased the serum cholesterol in LPK rats (Fig 10). Serum albumin, calcium and phosphate were similar in all groups (data not shown). Levels of sirolimus in whole blood were only detected in rats treated with sirolimus (Lewis+sirolimus: 11.8±6.2, LPK+sirolimus: 9.7±5.3 ng/ml).

#### Cystic Renal Disease

**Fig 11:**
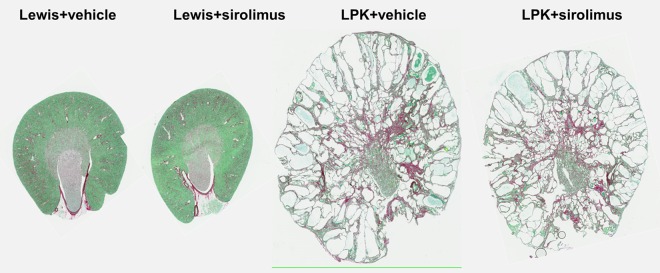
Effect of late initiation of sirolimus on renal histology at Week 20 in LPK and Lewis rats. Shown are representative whole-slide digital images of sections stained with Sirius-red from the experimental group. Sirolimus administration from weeks 10 to 20 reduced kidney size but did not alter the percentage cyst area and interstitial fibrosis in LPK rats.

At week 20, LPK rats had developed diffuse cystic renal disease characterised by gross collecting duct and distal nephron ectasia associated with interstitial disease (Fig 11). Although there was a partial reduction (by 20.0%) in the cross-sectional area of the kidney section in the LPK+sirolimus group compared to LPK+vehicle, the percentage cyst area was not altered (Table 3). In contrast to Study 2, the delayed commencement of sirolimus also did not alter renal cell proliferation, as assessed by the number of BrdU+ or Ki67+ cells (Table 3). In addition, the progression of interstitial inflammation and fibrosis (monocyte and myofibroblast accumulation) were also unaffected by sirolimus in LPK rats (Table 3).

**Table 3:** Effects of sirolimus on cystic renal disease at Week 20 in late-stage PKD. Data expressed as mean±SD; Abbrevations: BrdU, bromodeoxyuridine; αSMA, alpha-smooth muscle actin; SR, Sirius-Red; *P<0.05 when compared to Lewis+vehicle; #P<0.05 when compared to LPK+vehicle.

	Lewis+Vehicle	Lewis+Sirolimus	LPK+Vehicle	LPK+Sirolimus
	N = 5	N = 5	N = 10	N = 10
Kidney section area (mm^2^)	100±7	93±7	145±17*	116±9*#
Cystic area (%)	-	-	58.5±6.4	56.5±4.6
BrdU+ cells (%)	0.7±0.8	0.9±0.8	8.7±4.4*	10.0±4.4*
Ki67+ cells (%)	0.16 ± 0.09	0.18 ± 0.12	1.10 ± 0.62*	0.98 ± 0.46*
ED-1+ cells (%)	9.2±3.0	9.0±1.8	23.3±3.9*	20.7±4.5*
α-SMA+ cells (%)	8.1±2.7	6.9±2.9	26.3±7.1*	22.9±5.8*
Interstitial collagen (SR-positive) (%)	8.7±6	10.1±5	39.0±11.6*	34.5.0±9

#### Renal TORC1 and TORC2 activation

**Fig 12:**
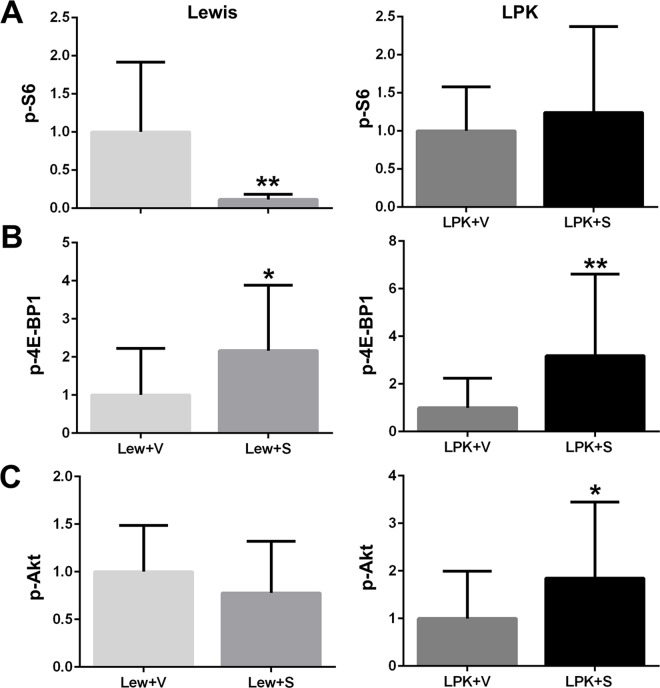
**Effect of late initiation of sirolimus on renal p-S6 (A), p-4E-BP1 (B) and p-Akt (C) as assessed by quantitative analysis of immunostaining.** Data (mean±SD) are expressed as the fold-change over the average for vehicle-treated animals; *p<0.05 vs. vehicle; **p<0.01 vs. vehicle, n = 5 per group for Lewis rats and n = 10 per group for LPK rats.

**Fig 13:**
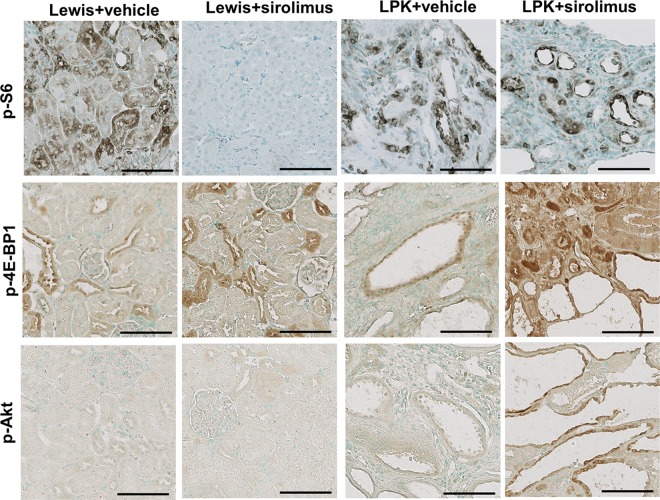
**Effect of late initiation of sirolimus on renal p-S6 (A), p-4E-BP1 (B) and p-Akt.** Representative photomicrographs of immunostaining for p-S6, p-4EBP1 and p-Akt in the kidney at week 20 from Lewis and LPK rats treated from week 10 until week 20. Scale bar = 100μm.

In Lewis rats, treatment with sirolimus from weeks 10 to 20, led to a marked decrease in the renal expression of p-S6 and an increase in p-4E-BP1 (Figs 12 and 13), whereas there was no change in p-Akt (Fig 12). In LPK rats, sirolimus did not affect renal p-S6 expression but increased p-4E-BP1 and p-Akt compared to LPK+vehicle (Figs 12 and 13).

#### Renal pro-inflammatory gene expression and NF-κB activation

**Fig 14:**
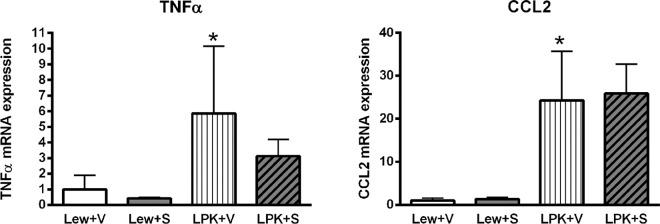
Effect of late initiation of sirolimus on NF-κB-dependent proinflammatory gene expression (*TNFα* and *CCL2*) in Lewis and LPK rats at Week 20 in Study 3. The mRNA expression is shown as the target gene corrected for GAPDH, and expressed as a fold-change over Lewis+V. Data are expressed as mean±SD; *p<0.05 vs. Lewis+vehicle (V); n = 5 per group for Lewis rats and n = 10 per group for LPK rats.

TNF**α** mRNA was higher in vehicle-treated LPK rats compared to the Lewis group (P<0.05, Fig 14). There were no significant differences in *TNF***α** between Lewis+vehicle and Lewis+sirolimus (p = 0.70) or between LPK+vehicle and LPK+sirolimus (p = 0.12). *CCL2* expression was higher in vehicle-treated LPK rats compared to the Lewis group (p<0.05, Fig 14). Sirolimus treatment initiated at late-stage disease also did not significantly alter *CCL2* expression in Lewis or in LPK rats (Fig 14). NF-κB activation (p65-DNA binding activity) was increased in Lewis rats receiving sirolimus compared to the vehicle group (Lewis+vehicle: 1.0±0.03, Lewis+sirolimus: 1.3±0.2; P = 0.0495). This increase was also noted in LPK rats receiving sirolimus (LPK+vehicle: 0.8±0.2, LPK+sirolimus: 0.9±0.1; P = 0.0460). There was no significant effect of sirolimus on phosphorylated p105 immunostaining (S5 Fig).

### Effect of sirolimus on ultrastructural abnormalities in renal cilia in LPK rats

**Fig 15:**
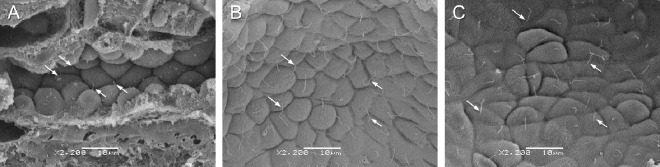
Effect of sirolimus on ultrastructural abnormalities in renal cilia in LPK rats. Shown are representative SEM images of renal epithelial cells highlighting cilia (arrows) from Lewis untreated control (A: age 10 weeks) and LPK treated with vehicle (B: age 20 weeks, Study 3) or sirolimus (C: age 20 weeks, Study 3). Scale bars in panels A to C are each 10**μ**m with images all taken at the same magnification (x 2200).

In Study 2 (treatment from weeks 3 to 10 weeks of age with sirolimus) tissue was analysed from LPK+vehicle (n = 3) and LPK+sirolimus (n = 3) groups. For the Lewis+vehicle and Lewis+sirolimus groups kidney tissue was not suitable for SEM processing and so kidneys were collected and analysed from an additional two Lewis control (untreated). In Study 3 (treatment from 10 to 20 weeks of age with sirolimus), kidneys were collected and analysed from all groups: Lewis+vehicle (n = 3), Lewis+sirolimus (n = 3), LPK+vehicle (n = 4) and LPK+sirolimus (n = 4). Representative SEM images of cilia are shown in Fig 15. Lewis kidneys at age 10 and 20 weeks showed normal morphology, with cilia present on the majority of cells (excluding intercalated cells of collecting tubules) as short projections into the lumens of the tubules. In the LPK rat, cilia were often long, tangled, screwed, with branches or knots. Occasionally there were multiple cilia per cell. However, by qualitative analysis, treatment with sirolimus did not alter cilia length or abnormal cilia morphology in either the early or late initiation study (Fig 15).

### Effect of sirolimus on cardiovascular disease in LPK rats

**Fig 16:**
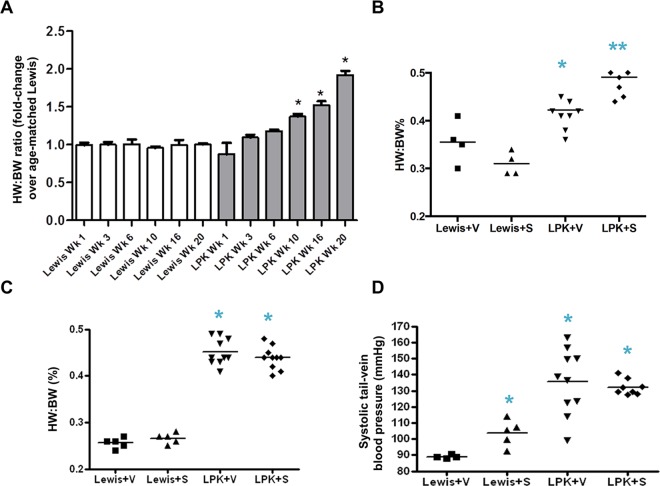
Effect of sirolimus on cardiovascular disease in LPK rats. Shown are changes in the heart to body weight (HW:BW) ratio in Studies 1, 2 and 3 (Panels A, B and C respectively) and systolic tail arterial blood pressure in Study 3 (Panel D). Data are expressed as mean±SD; *P<0.05 vs. Lewis+Vehicle (S); #P<0.05 vs. LPK+vehicle; n = 5 per group for Lewis rats and n = 10 per group for LPK rats.

In Study 1, over the course of 20 weeks, the heart to body weight ratio of LPK rats increased progressively compared to age-matched Lewis rats, becoming statistically different at weeks 10, 16 and 20 (Fig 16). In Study 2, sirolimus caused a slight but significant increase in the heart to body weight ratio in LPK rats (Fig 16). In Study 3, sirolimus did not alter the increase in cardiac enlargement (Fig 16). Systolic tail arterial blood pressure was only assessed in Study 3, and it was 1.5 times higher in the LPK+vehicle group compared to Lewis+vehicle rats (Fig 16). Sirolimus treatment increased systolic blood pressure in Lewis rats but did not affect the LPK group (Fig 16).

**Fig 17:**
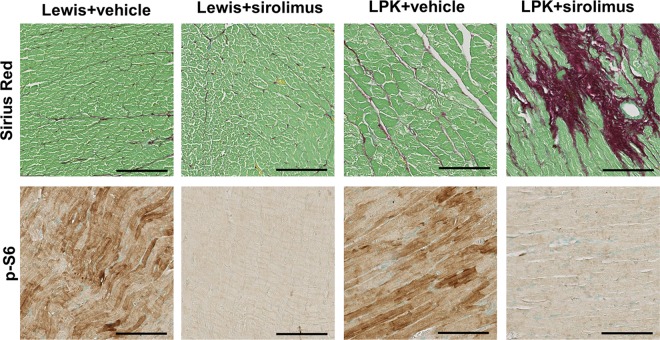
Effect of late initiation of sirolimus on cardiac histology at week 20. Shown are representative photomicrographs of heart tissue for interstitial collagen deposition (Sirius Red, upper panels) and p-S6 (lower panels) in Lewis and LPK rats at week 20 (Study 3). Scale bar = 100μm.

Further analysis of cardiac histology was undertaken in a subset of randomly selected animals (Lewis, n = 3 per group; LPK, n = 4 per group from Study 2 and 3) (Table 4 and Table 5). Qualitative assessment of PAS-stained coronal heart sections revealed no observable differences in cardiac histology among the groups in either study (data not shown). In Study 2, sirolimus reduced interstitial collagen deposition in the hearts of Lewis rats whereas there was no effect in LPK rats (Table 4). In contrast, in Study 3 sirolimus increased interstitial collagen deposition in LPK rats (Table 5 and Fig 17). Quantitative analysis showed that sirolimus markedly reduced p-S6 in both Lewis and LPK groups in Study 2 and Study 3 (Tables 4 and 5, Fig 17). Cardiac p-Akt was elevated in LPK rats compared to Lewis rats in Study 2, but was not altered by sirolimus in either study (Tables 4 and 5). Taken together, these data suggest that the inhibition of TORC1 activation in cardiac tissue in this model of PKD does not favourably alter the progression of cardiovascular disease and may be associated with adverse effects.

**Table 4:** Effect of sirolimus on cardiac disease at Week 10 in early-stage PKD. Data expressed as mean±SD; *P<0.05 when compared to Lewis+vehicle; **P<0.01 when compared to Lewis+vehicle; #P<0.05 when compared to LPK+vehicle; ## P<0.01 when compared to LPK+vehicle.

	Lewis+vehicle	Lewis+sirolimus	LPK+vehicle	LPK+sirolimus
	N = 3	N = 3	N = 4	N = 4
Cardiac interstitial collagen (SR) (%)	1.19 ± 0.42	0.77 ± 0.28**	1.40 ± 0.73	1.51 ± 2.15
Cardiac p-S6 (%)	1.27 ± 1.20	0.008 ± 0.008**	1.80 ± 2.17	0.036 ± 0.037##
Cardiac p-Akt (%)	0.46 ± 0.21	0.31 ± 0.29	2.25 ± 2.21*	1.38 ± 2.18

**Table 5:** Effect of sirolimus on cardiac disease at Week 20 in late-stage PKD. Data expressed as mean±SD; *P<0.05 when compared to Lewis+vehicle; **P<0.01 when compared to Lewis+vehicle; #P<0.05 when compared to LPK+vehicle; ## P<0.01 when compared to LPK+vehicle.

	Lewis+vehicle	Lewis+sirolimus	LPK+vehicle	LPK+sirolimus
	N = 3	N = 3	N = 4	N = 4
Cardiac interstitial collagen (SR) (%)	4.35 ± 0.97	6.03 ± 5.82	3.43 ± 1.08*	9.28 ± 6.93##
Cardiac p-S6 (%)	6.37 ± 6.30	0.09 ± 0.17**	5.40 ± 4.56	0.05 ± 0.05##
Cardiac p-Akt (%)	0.36 ± 0.27	0.75 ± 1.03	0.55 ± 0.33	0.63 ± 0.65

## Discussion

In experimental models of PKD, the inhibition of TORC1 has been associated with reduced cyst growth in the kidney [7], [12], [5], [28], [8]. However, these results have not been reproducible in human clinical trials of ADPKD [9], [10]. The aim of this study was to determine whether the timing of sirolimus initiation (early or late stage) influences its therapeutic efficacy in PKD. The LPK rat, which is a stable chronic hypertensive model with robust changes of cystic renal disease, allowed the investigation of the therapeutic effects of sirolimus in different phases of disease [19, 20]. Treatment of animals from week 3 to 10 permitted the preventative effects of sirolimus on kidney enlargement to be assessed, whereas therapy from weeks 10 to 20 determined whether TORC1 inhibition has nephroprotective effects when kidney size is near maximum.

The most important finding of this study is that the relative treatment effect of sirolimus, as assessed by the rate of kidney enlargement (expressed as the percentage increase in TKV per week) in serial MRI imaging, was similar regardless of the phase of drug commencement (that is, 78.2 vs.71.8% in Studies 2 and 3 respectively). In contrast, the absolute effect of sirolimus on kidney enlargement (as assessed by the two kidney to body weight ratio) was greatest in Study 2 (that is 63% vs. 22%, in Studies 2 and 3 respectively), most likely because the kidney size was smaller at the time of commencing treatment. Overall, these results were contrary to our initial hypothesis, as in previous studies we showed that the intensity of proliferation is highest week 3 in LPK rats [13], and therefore surmised that all of the treatment effects of sirolimus on kidney enlargement might be largely restricted to when treatment was commenced in the early phase of disease.

One of the strengths of the current study is that it has been conducted in a rat model (rather than in mice), which allowed for the accurate determination of TKV in serial MR imaging as well as the harvest of larger volume of serum and kidney tissue for analysis of renal function as well as detailed histopathology. So far, only a few preclinical studies have examined the stage-specific effects of sirolimus in PKD and all have been undertaken in mouse models. Novalic *et al*. demonstrated that in iKsp-*Pkd1*^del^ mice, the early-stage initiation of sirolimus significantly decreased the kidney to body weight ratio and cystic index, whereas late-stage initiation altered neither parameter, even when administered at a higher dose [18]. In contrast in the same manuscript, Novalic *et al*. found that in *Pkd1*^nl,nl^ mice, the kidney to body weight ratio and cystic index were significantly reduced by high-dose sirolimus when commenced late [18]. Interestingly, Gattone *et al*. showed that late initiation of sirolimus in *pcy* mice (an *NPHP3* ortholog) reduced kidney to body weight ratio and cyst enlargement, while early initiation did not [8]. The different results in these two studies are probably due to phenotypic and genotypic differences in the animal models.

The ‘regression’ of cystic renal disease has received little attention in PKD in comparison to other types of chronic kidney disease (CKD) [29]. In PKD, cyst regression can be arbitrarily defined as a reduction in renal cyst volume and interstitial injury to less than before treatment was commenced, and should be differentiated from spontaneous rupture and dissolution of renal cysts due to mechanical injury [30]. This study did not find any evidence for renal cyst regression as assessed by histological measurement of percentage cyst area or in serial MR imaging. *In vivo* studies of sirolimus have produced varying findings regarding its effect on the regression of cystic disease. Renal cyst regression with sirolimus has been documented to occur in *pcy* mice [14] and *Pkd1* conditional null mice [7]. Our findings suggest that the regression of cystic renal disease is not a universal phenomenon of TORC1 inhibition in PKD, and at the very least drug therapy must be initiated at the earliest stages of kidney enlargement in order to gain the maximal absolute lifetime benefit on TKV reduction.

Our study also highlights the complex relationship between changes in TKV and renal function with TORC1 inhibition. Despite the marked attenuation in kidney enlargement in the early-treatment study, the significant decline in renal function was not altered. In contrast, late treatment led to a smaller change in TKV compared to early treatment, and was associated with a marginal improvement in renal function. The lack of correlation between renal function and kidney size has also been observed in the natural history of human ADPKD, where it is common for patients to have a normal eGFR despite a large TKV, since the remaining functional glomeruli compensate for the decreased filtrating ability of the dysfunctional nephrons [31]. Our results are similar to those of the clinical trial of everolimus by Walz *et al*., which demonstrated a decrease in TKV that was not accompanied by improvement in eGFR in ADPKD patients with Stage 2–3 CKD [10]. These observations suggest that renal function is also determined by factors other than TKV, such as interstitial fibrosis [32], as indicated in previous studies of animal models [13]. In addition, since sirolimus did not alter the cystic microarchitecture, the degree of physical obstruction and compression exhibited by the cysts on the surrounding nephrons also probably explains the lack of improvement in renal function. On the other hand, it is plausible that the sirolimus-induced reduction in TKV (in isolation from any changes in renal function and fibrosis) may provide some benefit over non-treatment, by reducing the risk of lower back discomfort and early satiety due to kidney enlargement [33]. Furthermore, in humans vasopressin receptor antagonists reduced TKV in parallel with slowing the decline of renal function in ADPKD, suggesting that other off-target and unknown effects of drugs that reduce TKV may also be important on their net effect on renal disease progression in ADPKD [34].

The effects of sirolimus on renal function in LPK and Lewis rats observed in our study also deserve specific discussion. To summarise, in LPK rats treatment with sirolimus during the early phase of disease (weeks 3 to 10) did not alter the progression of renal function, whereast treatment from weeks 10 to 20, partially attenuated the increase in serum creatinine and also the reduction in endogenous creatinine clearance in LPK rats. These results conflict with the findings in Lewis rats, where in Study 2, sirolimus reduced the creatinine clearance, and similarly in Study 3, it increased the serum creatinine and reduced the creatinine clearance. Therefore, chronic treatment with sirolimus (at the doses used in this study) reduced renal function in healthy rats [as reported also by other investigators [35]] but was moderately renoprotective in LPK rats. The differential effects on renal function in health and disease in this setting, is most likely explained, by the ability of sirolimus to reduce the rate of renal cyst growth in LPK rats and possibly also attenuation of glomerular hypertrophy associated with chronic injury [36].

Sirolimus has also been associated with proteinuria in renal transplant patients [37], [38], [39], and in clinical trials in ADPKD [9], [40]. Increased proteinuria associated with sirolimus has been hypothesised to be due to changes in glomerular permeability and vascular endothelial growth factor expression [41]. In the current study, we found no evidence that sirolimus worsens proteinuria in rats with established renal impairment due to PKD. In previous preclinical studies of PKD, TORC1 inhibition also has not been associated with adverse effects on proteinuria [42], [43], suggesting that susceptibility to this problem might differ between humans and animals.

There was a noticeable disparity between the effects of early and late sirolimus treatment on renal histopathology. Although the cystic microarchitecture was not altered by sirolimus at either disease-stage, there was a decrease in renal cell proliferation following early treatment in LPK rats (Study 2), whereas no change was seen in the late study (Study 3). This is in keeping with previous data that renal cell proliferation is time-dependant in LPK rats and peaks at week 3 [13]. Consistent with these observations, the absolute kidney section area (mm^2^) and percentage cystic area were both reduced by sirolimus in Study 2. In Study 3, sirolimus reduced the kidney section area but not the percentage cystic area. The reason for the discrepancy between the two studies is probably because that the reduction in kidney enlargement was more modest in Study 3 and that renal cystic disease in LPK rats is diffuse and very advanced at Week 20. Therefore, when the absolute value for cyst area was expressed as a percentage relative to the total kidney section area, no apparent change was detected.

The current study provides a detailed analysis of the renal histological effects of sirolimus in tubulointerstitial disease associated with PKD. In this regard, other investigators have suggested that attenuating renal interstitial fibrosis is an additional therapeutic approach to reducing the renal progression of PKD [44]. Previous studies have shown that sirolimus attenuates interstitial inflammation/fibrosis in models of chronic renal injury [15] but the effects in PKD have not been well defined. The data in the current manuscript indicates that sirolimus attenuates tubulointerstitial inflammation and fibrosis when commenced during early-stage disease but that this protective effect is no longer present if treatment is initiated when disease is established (Study 3).

In this paper we evaluated the renal expression of the down-stream targets of TORC1 and TORC2 activation to determine if there were disease-specific changes in their level of expression, and to confirm the molecular effects of sirolimus. Our study demonstrated that the renal expression of p-S6 (produced following TORC1 activation) fluctuated and was significantly increased in LPK rats only at postnatal week 3, coinciding with the time of peak renal cell proliferation in this model [13]. Sirolimus forms a complex with FK-binding protein (FKBP12) in the cytoplasm, which subsequently binds and inhibits TORC1, preventing the phosphorylation of S6 [45], [4]. As expected, early treatment with sirolimus reduced p-S6 staining in both LPK and Lewis rats [46], and this coincided with the significant reduction in TKV in LPK kidneys. In Study 3, late sirolimus treatment reduced p-S6 in Lewis animals but interestingly not in LPK rats. This finding contrasts with Novalic *et al*. who demonstrated suppression of p-S6 with both early and late sirolimus initiation [18]. That cardiac p-S6 expression was similarly reduced by sirolimus in Lewis and LPK animals in both Study 2 and Study 3, suggests that there may be resistance to the suppression of TORC1 in the kidney in late-stage PKD, for reasons that are not yet clear. Of course, a limitation is that the tissue expression of p-S6 has been assessed only at the final timepoint, and it cannot be excluded if p-S6 was reduced at an earlier timepoint in Study 3.

With regard to the other down-stream targets of TORC1 and TORC2, data from Study 1 showed that the renal expression of p-4E-BP1 remained stable in LPK rats whereas p-Akt was markedly increased in comparison to Lewis rats at all timepoints. The phosphorylation of 4E-BP1 (downstream of TORC1), is also known to be blocked by sirolimus [47]. However, our study found that p-4E-BP1 was increased by sirolimus in LPK kidneys in both early and late studies. Jiang *et al*. similarly observed that 4E-BP1 did not decrease following sirolimus treatment in rat liver tissue following partial hepatectomy, and remarked that kinases other than TORC1, might be responsible for phosphorylating 4E-BP1 [48]. The protein Akt lies upstream of TORC1 and its phosphorylation leads to subsequent mTOR activation [4]. In agreement with previous studies [49], we found that renal p-Akt expression was markedly elevated in LPK animals at all timepoints, indicating that TORC2 activity is upregulated in cystic renal disease [28]. Given its mechanism of action, we were not surprised that renal p-Akt in LPK rats was unaltered by sirolimus in Study 2. However, in Study 3, we observed sirolimus exacerbated renal p-Akt expression and, of relevance, this has also been noted by others [50]. In cardiac tissue, p-Akt was unaffected by sirolimus in LPK rats. Our study was not designed to evaluate the functional significance of the changes in p-4EBP1 and p-Akt nor the effects of sirolimus on other mitogenic pathways (such as ERK), and further experiments are needed to address these questions, perhaps through an *in vitro* approach [51].

Although a relationship between mTOR and NF-κB has been alluded to in the literature, studies have demonstrated varying results regarding the effect of sirolimus on NF-κB signaling [52], [53], [54], and it appears that the modulatory activities may differ according to cell type and pathological state [55]. While these studies indicate that Akt acts as a key intersection point between the mTOR and NF-κB pathways, it is unclear whether NF-κB is under the control of mTOR or vice versa. Given that evidence for both phenomena has been demonstrated, it is probable that mTOR and NF-κB mutually regulate each other’s activity in an interchangeable manner [56], [57], [58]. We hypothesised that sirolimus would negatively regulate NF-κB via mTOR in PKD, but found that it did not alter the renal expression of the p65 DNA binding activity, p-p105, or NF-κB-dependent genes *TNFα* and *CCL2*. A previous study in LPK rats demonstrated that renal *TNFα* and *CCL2* are elevated in the late stages of disease (week 10 to 20) [22], and our current data shows that sirolimus was ineffective in suppressing transcription these genes. Further *in vitro* studies in cystic renal cells are required to prove a direct linkage between NF-κB and mTOR.

Abnormalities in the morphology of primary cilia have been associated with renal cystic disease. Longer cilia have been reported also in the *cpk* mouse [59] and other animal models within the cystic renal disease spectrum, such as the *wpk* or *jck* mouse [60], [61], [62]. Cilium elongation is also linked to injury [63], [64], [65], and has been proposed by some researchers to be a consequence of cystogenesis. Alternatively, others have suggested that a primary defect in cilium assembly and maintenance is a key pathogenic mechanism of cystic kidney diseases. This study demonstrated longer cilia length in collecting and distal tubules of the kidney in LPK rats. Scanning electron microscopy allowed the assessment of anatomical abnormalities in the cilia. Unlike some other animal models of PKD (mks3) [60] and *orpk* mutant [66], multiple cilia per cell, while observed, were not common in our cohort, with the majority of epithelial cells showing a single cilium per cell, as in normal uninjured epithelium. Branching, screwing and knots were also observed in in our LPK cohort, however none of the above was found in Lewis controls. Tangling was commonly observed in LPK animals, and is most likely a result of increased cilia length.

Treatment with sirolimus did not alter ciliary abnormalities in either the early- or late-stage treatment groups. Importance of the changes in the morphology of the cilium is highlighted by the fact that mutations in proteins linked to this small cellular organelle are central in human and animal models of cystic kidney diseases [67] [68, 69]. Their functional significance for the maintenance of epithelial differentiation and proliferation during normal physiological division is well established [70], as is their role during the injury and repair process [63]. In PKD however, the implications of increased ciliary length remain unknown. Wang *et al*. [63] hypothesised that ciliary lengthening influences epithelium differentiation. In contrast, Verghese [64] proposed that lengthening is a response that increases sensory sensitivity and promotes differentiation, balancing increased dedifferentiation and facilitating redifferentiation that is required after injury [71]. Since sirolimus is a TORC1 inhibitor and is not known to directly modulate ciliary processes (e.g. intraflagellar transport), the lack of change in cilia length, number and morphology is consistent with the drug’s mechanism of action and also consistent with the hypothesis that it does not improve the loss of cellular differentiation in PKD.

Cardiovascular disease is the leading cause of mortality in ADPKD [72] and our study in LPK rats provided an opportunity to examine the effects of sirolimus on this disease process. Our data show that cardiovascular disease was not ameliorated by sirolimus in the LPK rat. On the contrary, the late administration of sirolimus led to an increase in systolic blood pressure in Lewis rats, and increased interstitial collagen deposition in LPK animals. Sirolimus has previously been found to increase blood pressure in rats, possibly via its effects on serotonin and modulating hemorheology [73]. In contrast to our findings, Zafar *et al*. demonstrated decreases in heart weight and mean arterial pressure with long-term sirolimus treatment in the Han:SPRD rat model of PKD [74]. Studies have also reported improvements in cardiac fibrosis following sirolimus treatment in other animal models of heart disease [75], [76]. Given that there are limited data on the impact of sirolimus on cardiac disease in experimental PKD, our results should be investigated further.

The results of the present study imply that drug dosage is probably one of the additional factors in differentiating between the efficacy of sirolimus in animal and human PKD studies. This view has also reached by others [18]. While human ADPKD patients experienced adverse effects (including mouth ulcers, diarrhoea, peripheral edema and hyperlipidemia) with 2 mg daily sirolimus [9], the drug has been generally considered to be well-tolerated in rodents at doses of 0.2 to 5mg/kg [8], [5], [12]. Recent studies have investigated folate-conjugated sirolimus in enhancing drug delivery to renal cells, thereby increasing drug exposure to at the target site while reducing systemic adverse effects [77]. Ravichandran *et al*. have demonstrated that novel methods of mTOR inhibition, including mTOR kinase inhibitors and mTOR anti-sense oligonucleotide, attenuated kidney growth and renal function in murine PKD models [78], [79]. mTOR kinase inhibitors may also be less likely to induce leukopenia compared to TORC1 inhibitors [80]. While these approaches might minimise the systemic adverse effects associate with TORC1 inhibition, their differential efficacy in early- and late-stages of disease requires close scrutiny in preclinical studies, before clinical trials are undertaken.

## Conclusions

The present study demonstrated that the absolute effect of sirolimus in reducing kidney size in PKD is dependent on the TKV at the time of drug initiation but that that the relative treatment efficacy (rate of TKV increase) is similar at all stages of disease. Moreover, the reduction in TKV with sirolimus was not accompanied by improvements in cyst microarchitecture, interstitial inflammation or fibrosis, and insufficient to modify the progression of renal dysfunction. These findings have two important translational implications: (i) the use TORC1 inhibitors in PKD should ideally be able to be initiated early and continued long-term, which sirolimus, in its present formulation and method of delivery, does not meet these criteria due to adverse events and toxicity in humans; and (ii) a multi-pronged approach, encompassing strategies to reduce cell proliferation, promote cellular differentiation and reduce interstitial disease in conjunction with cardioprotection [81] and cyst-diminishment, will be required to effectively prevent kidney failure and mortality due to PKD [82].

## Supporting Information

S1 File Supplementary Methods. (DOCX)

S1 Fig Setup for MRI scanning using a clinical 3 Tesla scanner. See S1 File for further details.
(TIF)

S2 Fig Method of kidney segmentation using 3D SLICER. See S1 File for further details
(TIF)

S3 Fig Effect of early initiation of sirolimus on the renal expression of p-p105 in the experimental groups. In Lewis rats, there was moderate p-p105 staining in collecting ducts of the inner medulla and in tubular epithelia of the medullary rays. Lewis cortices displayed weak background staining, with moderate staining in the epithelium of distal tubules. Large positively stained cells were present in the renal pelvis. In LPK rats, p-p105 was present in cystic epithelial cells of the outer medulla and cortex, and in the epithelia of the inner medullary tubules. Large positive cells were also observed in the renal pelvis of LPK rats (not shown). There was no observable alteration in P-p105 staining with sirolimus treatment in Lewis or LPK. Scale bar = 100μm.
(TIF)

S4 Fig Sirolimus does not improve cystic micro-architecture on magnetic resonance imaging. High-power magnified sagittal and axial views of MR images of LPK animals treated with either vehicle or sirolimus at week 17, showing that although, TKV was reduced, abnormal cystic tubular dilatation and loss of corticomedullary differentiation remained abnormal with sirolimus treatment.
(TIF)

S5 Fig Effect of late initiation of sirolimus on the renal expression of p-p105 in the experimental groups. Lewis kidneys displayed moderate p-p105 staining in the inner medulla and weak cortical staining. LPK kidneys showed moderate p-p105 staining in cortical and outer medullary CECs, and moderate staining in dilated tubules of the inner medulla. Of note, there were occasional deposits of positive interstitial cells, (which were not observed in Study 2). However, similar to the early sirolimus study, large positive cells were observed in the renal pelvis of Lewis and LPK animals. Qualitative assessment of whole slides indicated that sirolimus treatment did not change the pattern or degree of p-p105 staining in either LPK or Lewis kidneys.
(TIF)
