# Supplementary material for: Effects of TORC1 Inhibition during the Early and Established Phases of Polycystic Kidney Disease
Source: PLoS One. 2016 Oct 10;11(10):e0164193. doi: 10.1371/journal.pone.0164193 (PMC5056751; doi:10.1371/journal.pone.0164193)
Supplement: S1 File — (DOCX) [file pone.0164193.s001.docx]

# Supporting Information

**SUPPLEMENTARY METHODS**

**MRI technique**

For MRI, we developed a technique to image kidneys in anaesthetised rats using a clinical 3T MRI scanner (S1 Fig). Prior to the scan, rats were given a restricted amount of food (10g per rat) for 16 hours, since in pilot studies we found that that gastric distension obscured renal images in non-fasted animals. Prior to the MRI, rats were anaesthetised by isoflurane inhalation (5%) using a vapouriser and inhalation box (S1 Fig). Following sedation, animals were gently secured on a small padded bed using micropore tape and crepe bandages, and anaesthesia was maintained with isoflurane (2%) by attaching tubing from the vaporiser to a 50 ml syringe (with the plunger removed), which was placed over the face of the animal (S1 Fig). The vaporiser was located outside of the MRI room and attached to the syringe through extension tubing. The procedure to perform the scan generally took between 30-45 minutes for each animal, and following withdrawal of the isoflurane, animals gained consciousness within 5 minutes and made a complete recovery.

The MRI was performed using a cylindrical Mayo BC-10 transmit/receive RF coil (Mayo Clinic Medical Devices) clinically used for wrist imaging on a GE SIGNA Twinspeed HDxt 3T MR system. All animals were scanned tail first using a coronal and axial T2-W 3D FIESTA sequence. The scan parameters for the two sequences were as follows - Coronal: FOV=10mm, Phase FOV=1 TE/TR= 4.1/12.1 msec, Flip angle=45°, 352*256 acq. matrix, R/L Freq direction, 32 locations per slab with effective slice thickness 0.8mm, 2 NEX, Acquisition time= 6min 44sec ; Axial: FOV=9mm, Phase FOV=0.7 TE/TR= 4.3/13.7 msec, Flip angle=45°, 352*256 acq. matrix, R/L Freq direction, 32 locations per slab with effective slice thickness 0.8mm, 2 NEX, Acquisition time = 5min 26sec.

Both the left and right kidneys were segmented from the coronal volume acquisition. Segmentation was performed using a semiautomatic fast marching segmentation algorithm implemented using the freely available 3D SLICER program ([www.slicer.org](http://www.slicer.org)) [1, 2] (S2 Fig). The Total Kidney Volume (TKV) was computed from the segmented images and the assessor (K.G. Schwensen) was blinded to the categorisation of the animals.

**Assessment of κB-dependent inflammatory genes**

To determine the expression of **κ**B-dependent pro-inflammatory genes relevant to PKD (*TNF***α** and *CCL2*), RNA was extracted from 100 mg of snap-frozen kidney tissue using the RNeasy Mini Kit (Qiagen, Venlo, Limburg, Netherlands). RNA was reverse-transcribed into cDNA (SuperScript III First-Strand Synthesis System, Thermo Fisher Scientific, Waltham, MA, USA) using oligo(dT) and dNTP, and using the cDNA Synthesis Mastermix according to manufacturer’s instructions. Real-time quantitative PCR was performed using Platinum SYBR Green qPCR SuperMix-UDG (Thermo) on a BioRad CFX96 machine (Bio-Rad Laboratories, Hercules, CA, USA). The sequence for the PCR primers were as follows: *TNFα* (forward: 5’GTC GTA GCA AAC CAC CAA GC 3’, reverse: 5’ TGT GGG TGA GGA GCA CAT AG 3’) [3]; *CCL2* (forward: 5’AGC CCA GAA ACC AGC CAA CTC 3’, reverse: 5’ GCC GAC TCA TTG GGA TCA TCT T 3’) [4]; and GAPDH (forward: 5’ GAA CAT CAT CCC TGC ATC CA 3’), reverse: 5’ CCA GTG AGC TTC CCG TTC A 3’) [5]. PCR parameters were 95°C for 2 minutes and 95°C for 15 seconds, 60°C for 30 seconds, and 72°C for 30 seconds for 40 cycles; melting temperature was measured between 65°C and 95°C. Data were analysed using CFX Manager software (v3.1.1517.0823, 2012 release, Bio-Rad). Gene expression was quantified using the ΔΔCT method. The mRNA of *TNFα* and *CCL2* in each sample was normalised to GAPDH mRNA.

**FIGURE LEGENDS FOR SUPPLEMENTARY FIGURES**

**S1 Fig.** **Setup for MRI scanning using a clinical 3 Tesla scanner.** See Supplementary Methods for further details.

**S2 Fig.** **Method of kidney segmentation using 3D SLICER**. See Supplementary Methods for further details

**S3 Fig. Effect of early initiation of sirolimus on the renal expression of p-p105 in the experimental groups.** In Lewis rats, there was moderate p-p105 staining in collecting ducts of the inner medulla and in tubular epithelia of the medullary rays. Lewis cortices displayed weak background staining, with moderate staining in the epithelium of distal tubules. Large positively stained cells were present in the renal pelvis. In LPK rats, p-p105 was present in cystic epithelial cells of the outer medulla and cortex, and in the epithelia of the inner medullary tubules. Large positive cells were also observed in the renal pelvis of LPK rats (not shown). There was no observable alteration in P-p105 staining with sirolimus treatment in Lewis or LPK. Scale bar = 100µm.

**S4 Fig**. **Sirolimus does not improve cystic micro-architecture on magnetic resonance imaging.**  High-power magnified sagittal and axial views of MR images of LPK animals treated with either vehicle or sirolimus at week 17, showing that although, TKV was reduced, abnormal cystic tubular dilatation and loss of corticomedullary differentiation remained abnormal with sirolimus treatment.

**S5 Fig. Effect of late initiation of sirolimus on the renal expression of p-p105 in the experimental groups.** Lewis kidneys displayed moderate p-p105 staining in the inner medulla and weak cortical staining. LPK kidneys showed moderate p-p105 staining in cortical and outer medullary CECs, and moderate staining in dilated tubules of the inner medulla. Of note, there were occasional deposits of positive interstitial cells, (which were not observed in Study 2). However, similar to the early sirolimus study, large positive cells were observed in the renal pelvis of Lewis and LPK animals. Qualitative assessment of whole slides indicated that sirolimus treatment did not change the pattern or degree of p-p105 staining in either LPK or Lewis kidneys.

# References

1. Rangan GK, Schwensen KG, Foster SL, Korgaonkar MS, Peduto A, Harris DC. Chronic effects of dietary vitamin D deficiency without increased calcium supplementation on the progression of experimental polycystic kidney disease. Am J Physiol Renal Physiol. 2013;305(4):F574-82. doi: 10.1152/ajprenal.00411.2012. PubMed PMID: 23698116.

2. Ta MH, Rao P, Korgaonkar M, Foster SF, Peduto A, Harris DC, et al. Pyrrolidine dithiocarbamate reduces the progression of total kidney volume and cyst enlargement in experimental polycystic kidney disease. Physiological reports. 2014;2(12):e12196. Epub 2014/12/17. doi: 10.14814/phy2.12196. PubMed PMID: 25501440.

3. Elks CM, Mariappan N, Haque M, Guggilam A, Majid DS, Francis J. Chronic NF-{kappa}B blockade reduces cytosolic and mitochondrial oxidative stress and attenuates renal injury and hypertension in SHR. Am J Physiol Renal Physiol. 2009;296(2):F298-305. doi: 10.1152/ajprenal.90628.2008. PubMed PMID: 19073636; PubMed Central PMCID: PMCPMC2643866.

4. Mu W, Ouyang X, Agarwal A, Zhang L, Long DA, Cruz PE, et al. IL-10 suppresses chemokines, inflammation, and fibrosis in a model of chronic renal disease. Journal of the American Society of Nephrology : JASN. 2005;16(12):3651-60. doi: 10.1681/ASN.2005030297. PubMed PMID: 16251240.

5. de Borst MH, van Timmeren MM, Vaidya VS, de Boer RA, van Dalen MB, Kramer AB, et al. Induction of kidney injury molecule-1 in homozygous Ren2 rats is attenuated by blockade of the renin-angiotensin system or p38 MAP kinase. Am J Physiol Renal Physiol. 2007;292(1):F313-20. doi: 10.1152/ajprenal.00180.2006. PubMed PMID: 16896183.
