# Supplementary figures and images for: Effects of TORC1 Inhibition during the Early and Established Phases of Polycystic Kidney Disease
Source: PLoS One. 2016 Oct 10;11(10):e0164193. doi: 10.1371/journal.pone.0164193 (PMC5056751; doi:10.1371/journal.pone.0164193)

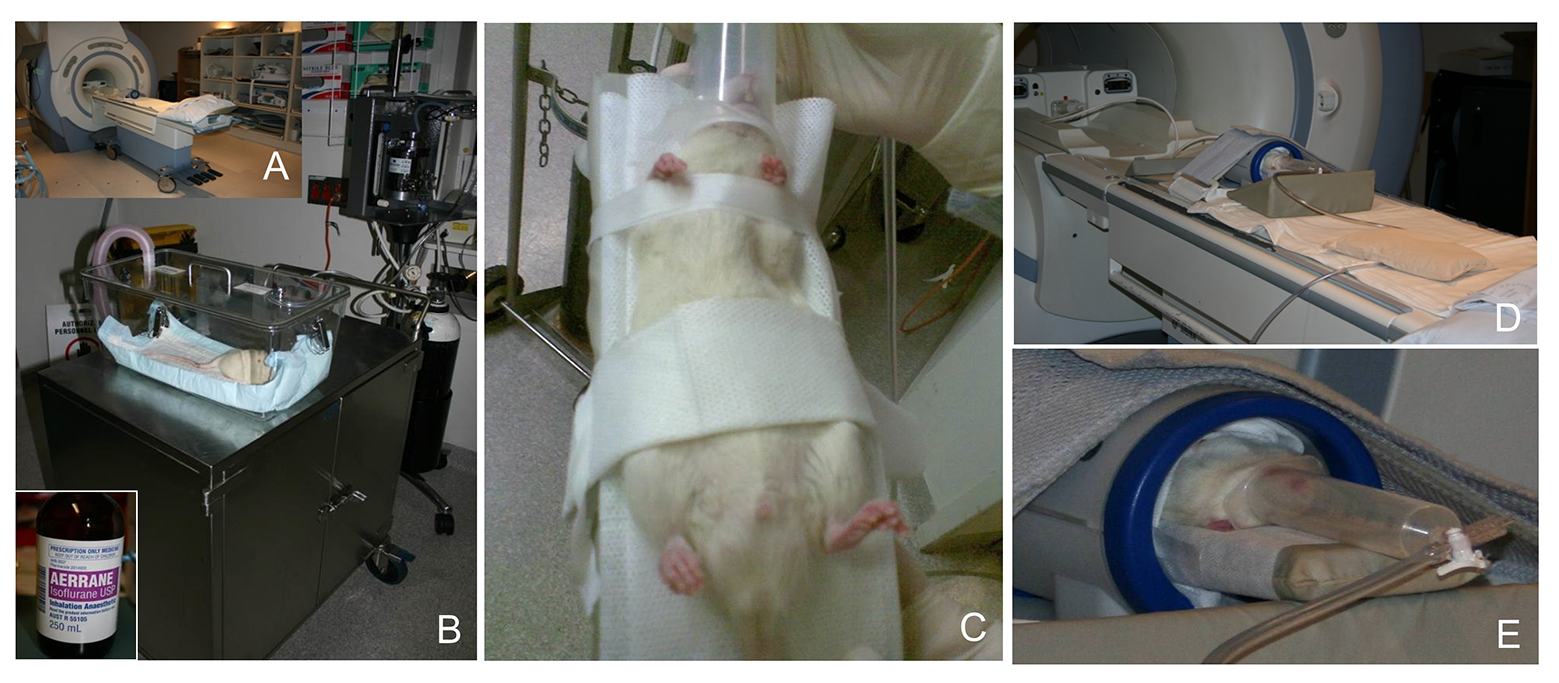

Supplement: S1 Fig — See S1 File for further details. (TIF) [file pone.0164193.s002.tif]

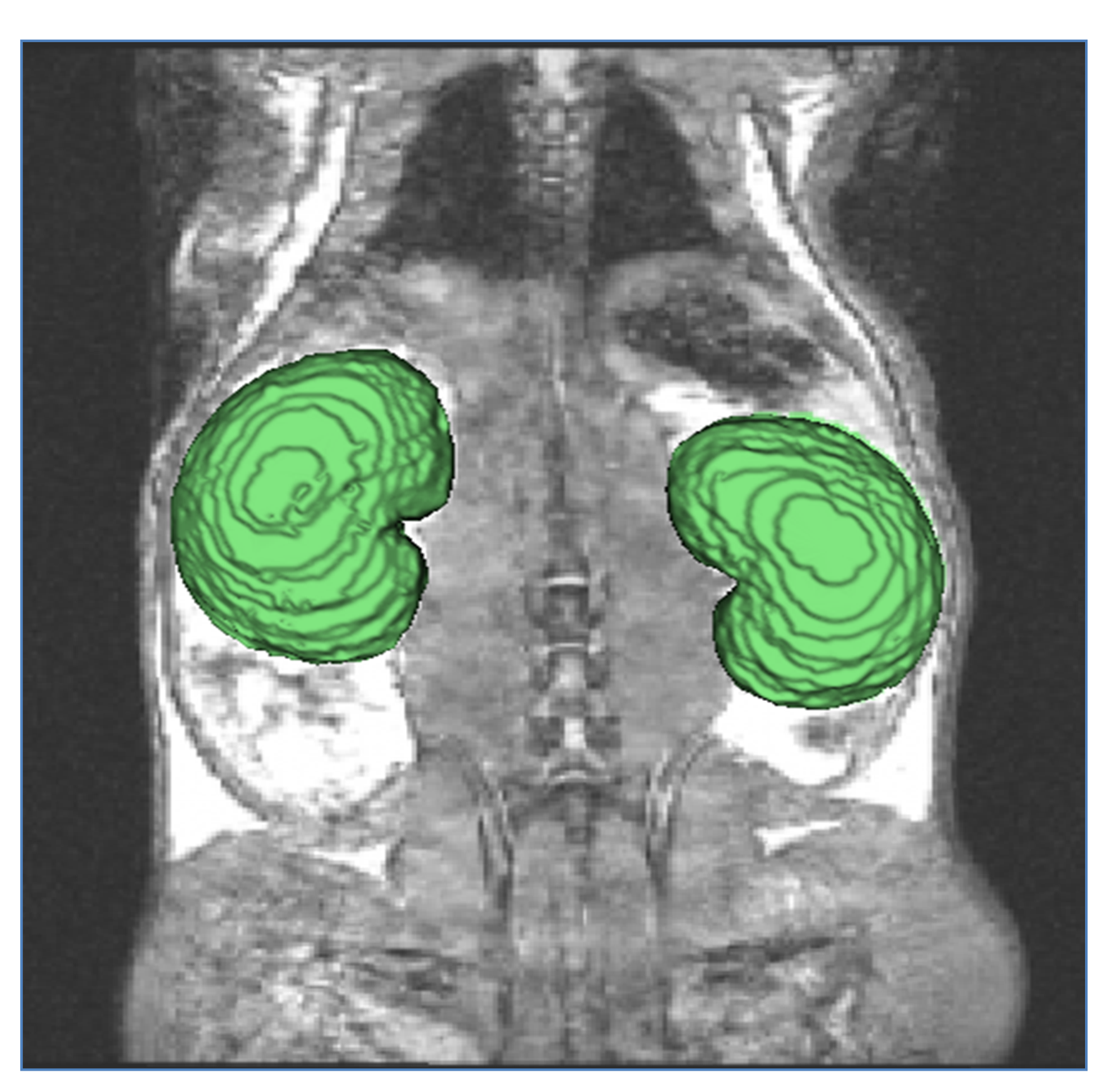

Supplement: S2 Fig — See S1 File for further details (TIF) [file pone.0164193.s003.tif]

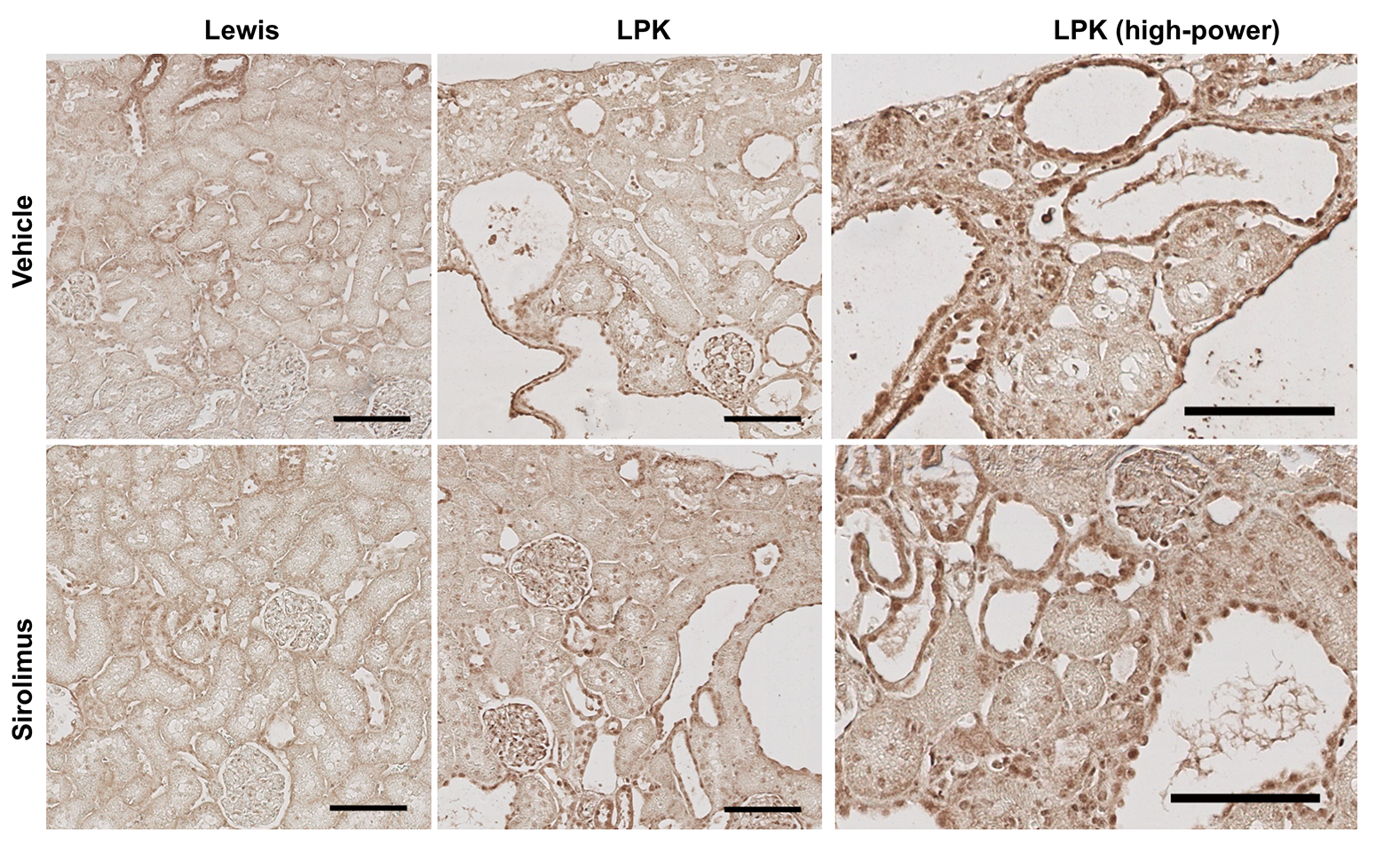

Supplement: S3 Fig — In Lewis rats, there was moderate p-p105 staining in collecting ducts of the inner medulla and in tubular epithelia of the medullary rays. Lewis cortices displayed weak background staining, with moderate staining in the epithelium of distal tubules. Large positively stained cells were present in the renal pelvis. In LPK rats, p-p105 was present in cystic epithelial cells of the outer medulla and cortex, and in the epithelia of the inner medullary tubules. Large positive cells were also observed in the renal pelvis of LPK rats (not shown). There was no observable alteration in P-p105 staining with sirolimus treatment in Lewis or LPK. Scale bar = 100μm. (TIF) [file pone.0164193.s004.tif]

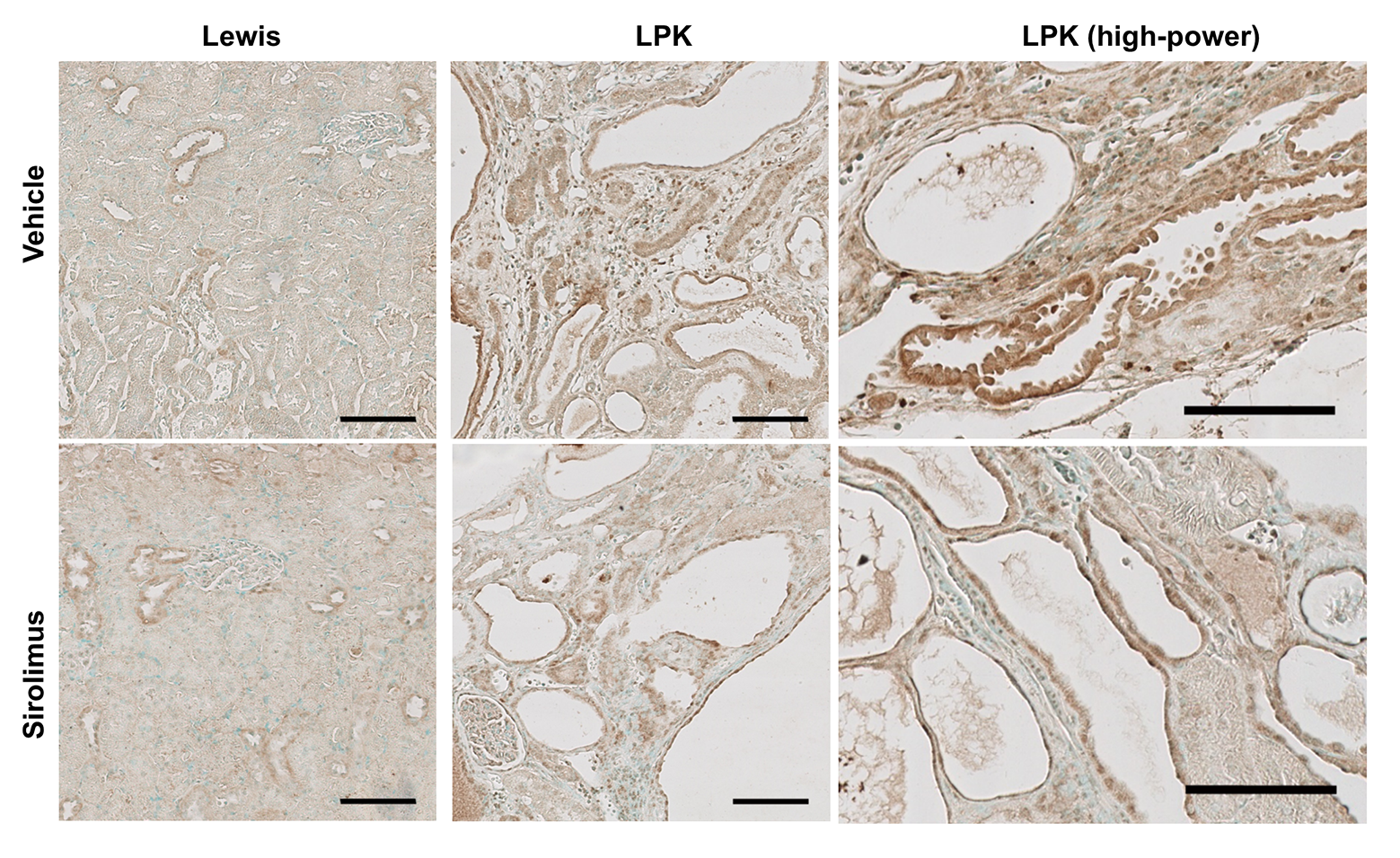

Supplement: S5 Fig — Lewis kidneys displayed moderate p-p105 staining in the inner medulla and weak cortical staining. LPK kidneys showed moderate p-p105 staining in cortical and outer medullary CECs, and moderate staining in dilated tubules of the inner medulla. Of note, there were occasional deposits of positive interstitial cells, (which were not observed in Study 2). However, similar to the early sirolimus study, large positive cells were observed in the renal pelvis of Lewis and LPK animals. Qualitative assessment of whole slides indicated that sirolimus treatment did not change the pattern or degree of p-p105 staining in either LPK or Lewis kidneys. (TIF) [file pone.0164193.s006.tif]
